# Unraveling the Role of Interfacial Charge Transfer on Photoactivity and Anomalous Luminescence Quenching of V_4_C_3_T*_*x*_*/Protonated g-C_3_N_4_ Heterostructures

**DOI:** 10.1021/acsami.4c19729

**Published:** 2025-03-10

**Authors:** Muhammad Abiyyu Kenichi Purbayanto, Madhurya Chandel, Michał Makowski, Muhammad Danang Birowosuto, Verónica Montes-García, Kaitlyn Prenger, Artur Ciesielski, Michael Naguib, Agnieszka Maria Jastrzębska

**Affiliations:** † Warsaw University of Technology, Faculty of Mechatronics, św. Andrzeja Boboli 8, 02-525 Warsaw, Poland; ‡ Łukasiewicz Research Network—PORT Polish Center for Technology Development, Stabłowicka 147, 54-066 Wrocław, Poland; § Université de Strasbourg, CNRS, ISIS, 8 allée Gaspard Monge, 67000 Strasbourg, France; ∥ Department of Physics and Engineering Physics, Tulane University, New Orleans, Louisiana 70118, United States; ⊥ Center for Advanced Technologies, Adam Mickiewicz University, Uniwersytetu Poznańskiego 10, 61-614 Poznań, Poland

**Keywords:** protonated g-C_3_N_4_, V_4_C_3_T*_*x*_*, MXenes, optoelectronics, heterostructures

## Abstract

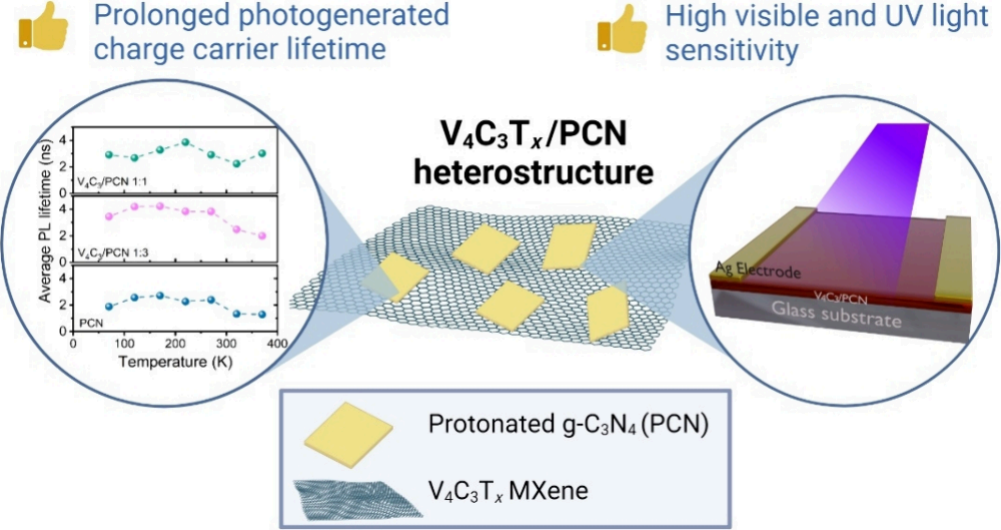

Two-dimensional van der Waals heterostructures with exotic quantum phenomena have garnered a huge surge in the field of optoelectronic devices. Herein, we report spectroscopic evidence of efficient interfacial charge transfers at the interface of a novel 2D/2D V_4_C_3_T*_*x*_* MXene/protonated g-C_3_N_4_ (PCN) heterostructured thin film, demonstrating robust photosensitivity and a large exciton activation energy of 139.5 meV. Through temperature-dependent photoluminescence (PL) and time-resolved PL spectroscopy, we unravel the photophysical mechanism driving efficient charge transfer and photosensitivity in V_4_C_3_T*_*x*_*/PCN heterostructures. These heterostructures exhibit superior photosensitivity to white and UV light compared with either PCN or V_4_C_3_T*_*x*_* pristine materials. Additionally, we observed significant PL quenching with unusual negative thermal quenching and extended charge carrier lifetime in the V_4_C_3_T*_*x*_*/PCN heterostructures across a broad temperature range of 70–370 K. Notably, at the elevated temperature of 370 K, the carrier lifetime was enhanced by more than 2-fold, making the heterostructures promising for optoelectronic applications. This work provides critical insight into the charge transfer mechanism between V_4_C_3_T*_*x*_* MXene and PCN, opening a new avenue for rationally designing g-C_3_N_4_-based heterostructures for highly photosensitive optoelectronic devices.

## 1 Introduction

Optoelectronic devices have catalyzed substantial technological advancements across various fields, including information, medicine, aerospace, defense, and environmental monitoring.^1^ The discovery of graphene has been instrumental in unlocking the potential of two-dimensional (2D) materials for use in optoelectronic applications, owing to their remarkable physical properties, such as pronounced quantum confinement effects.^2,3^ Moreover, the atomically thin structure of 2D materials facilitates easy integration into devices and ensures compatibility with current silicon photonic technology.^4^ Beyond graphene, a wide range of 2D layered materials has been identified, spanning from metallic to insulating types, such as transition-metal dichalcogenides (TMD), black phosphorus, transition-metal carbides/nitrides (MXenes), transition-metal borides (MBenes), hexagonal boron nitride (h-BN), and polymeric graphitic carbon nitride (g-C_3_N_4_).^5,6^ Among them, g-C_3_N_4_ has attracted significant attention as an optically active material, particularly following the seminal work by Wang et al., which demonstrated its potential as a metal-free photocatalyst operating under visible light irradiation.^7^ The use of g-C_3_N_4_ offers several advantages, including its intrinsic bandgap of 2.7 eV, high excitonic binding energy (∼328 meV), low cost, tunable emission properties, and sensitivity across the blue-to-green light spectrum.^8−11^ While much of the research on g-C_3_N_4_ has focused on its photocatalytic and photochemical applications, its optoelectrical properties offer substantial potential for exploration in next-generation optoelectronic devices.^9^ Moreover, despite its promising characteristics, only a few fundamental studies have explored the photophysics and carrier recombination processes in g-C_3_N_4_, which are crucial for the advancement of optoelectronic technologies.^8,9,12^ Additionally, owing to its layered van der Waals structure, g-C_3_N_4_ can be easily exfoliated into 2D nanosheets, considerably enhancing charge carrier density and quantum confinement.^13^

Despite its excellent optoelectrical properties, g-C_3_N_4_ faces several challenges, including rapid of electron–hole pair recombination, limited light absorption, poor electrical conductivity, and difficulties in postsynthesis processing due to its insolubility.^14,15^ To address these limitations, the fabrication of 2D/2D hybrid heterostructures has emerged as a highly effective strategy. Such heterostructures can significantly enhance charge transfer and separation through the built-in electrical field at the atomically thin interfaces.^16^ In this context, MXenes are considered as ideal building blocks for 2D/2D heterostructures because of their high electrical conductivity, excellent dispersibility, and strong optical absorption.^5,17^ In recent years, MXenes, a large family of transition-metal carbides, nitrides, and carbonitrides, have been shown to enhance the photocatalytic activity of g-C_3_N_4_. For example, hybridizing g-C_3_N_4_ with Ti_3_C_2_T*_*x*_* MXene has been reported to improve light absorption and simultaneously reduce charge recombination, as evidenced by an increase in the average fluorescence lifetime from 8.20 ns (for pristine g-C_3_N_4_) to 9.32 ns (in the g-C_3_N_4_/MXene heterostructure).^48^ Furthermore, a close interfacial connection was achieved through electrostatic interactions between positively charged protonated g-C_3_N_4_ (PCN) and negatively charged MXenes.^19,20^ The intimate contact between PCN and Ti_3_C_2_T*_*x*_* MXene leads to separation of photogenerated charge carrier at the interface and suppresses recombination of photogenerated electrons and holes.^20^ While extensive research has focused on g-C_3_N_4_/MXene heterostructures, particularly for photocatalytic applications, their fundamental optoelectronic properties and photophysics remain poorly understood. Recently, V_4_C_3_T*_*x*_* MXenes have emerged as a promising heterostructure component due to their larger interlayer spacing, superior saturable optical absorption, greater structural and thermal stability, abundance valence states (from V^2+^ to V^5+^), and richer surface chemistry compared to Ti_3_C_2_T*_*x*_*.^21−25^ Notably, the extinction coefficient of V_4_C_3_T*_*x*_* reaches 33.8 L g^–1^ cm^–1^ at 808 nm, much higher than Ti_3_C_2_T*_*x*_* (25.2 L g^–1^ cm^–1^), making it an excellent candidate for photon absorption.^26^

Building on recent advancements in the optoelectronic applications of g-C_3_N_4_ and V_4_C_3_T*_*x*_* MXenes, we have successfully fabricated an ultrathin 2D/2D V_4_C_3_T*_*x*_*/PCN heterostructure employing a straightforward electrostatic assembly method. We conducted an in-depth investigation of the photogenerated charge carrier behavior and excitonic dynamics of the heterostructure through temperature-dependent photoluminescence (PL) and time-resolved PL spectroscopy. To the best of our knowledge, this is the first study to explore photophysical phenomena in MXene/g-C_3_N_4_ heterostructures by using these characterization techniques. Interestingly, the optimized V_4_C_3_T*_*x*_*/PCN heterostructures demonstrated efficient separation of photogenerated charge carriers and extended exciton lifetime across a broad temperature range (70–370 K). This was highlighted by a 2.3-fold increase in PL lifetime compared to that of pristine PCN at 370 K. Furthermore, the heterostructure exhibited superior photosensitivity to white light compared to that of pristine V_4_C_3_T*_*x*_* or PCN. These findings may pave the way for the advancement of PCN-based heterostructures in optoelectronic devices.

## 2 Experimental Section

### 2.1 Materials

Hydrofluoric acid (HF, 48 wt %), hydrochloric acid (HCl, 35–38%), urea, Na_2_SO_4_ (99%), and isopropanol (99.5% purity) were acquired from Warchem. Tetramethylammonium hydroxide (TMAOH, 25 wt % aqueous solution), and Hellmanex III cleaning concentrate were purchased from Sigma-Aldrich. The glass substrate (15 × 15 mm size) was obtained from Chemland. The indium tin oxide (ITO)-coated glass substrate was purchased from 3D Nano, Poland. Double-distilled water (DDW) was used in this study.

### 2.2 Preparation of V_4_AlC_3_ MAX Phase

The V_4_AlC_3_ MAX phase was synthesized similarly to previously reported studies.^27^ In short, powders of vanadium (Alfa Aesar, −325 mesh, 99.5%), aluminum (Alfa Aesar, −325 mesh, 99%), and carbon (graphite, Alfa Aesar, APS 7–11 μm, 99%) were mixed in a ratio of 4.00:1.50:3.00 in an HDPE jar with 10 mm yttrium-stabilized zirconia balls in a Turbula T2F mixer for 3 h at ∼56 rpm. The mixed powders were pressed into 1 in. pellets of ∼10 g each, placed into an alumina crucible, and then heated in a SentroTech tube furnace. The heating cycle was 10 °C/min to 1000 °C and then 5 °C/min to 1500 °C, held at 1500 °C for 2 h, and then the furnace was allowed to cool to room temperature. All heating and cooling steps were performed under flowing argon. After being cooled, the pellets were crushed using a mortar and pestle and sieved to obtain −325 mesh particles.

### 2.3 Synthesis of V_4_C_3_T*_*x*_* MXene Nanoflakes

First, 500 mg of V_4_AlC_3_ was pretreated by stirring (100 rpm) in 5 mL of 9 M HCl. The pretreatment was done overnight to ensure the removal of oxide layers on the V_4_AlC_3_ surface. The treated V_4_AlC_3_ was washed with a repeating cycle (1500 RCF, 5 min) with double-distilled water (DDW) until pH ∼ 6. Next, V_4_AlC_3_ was added slowly into 48 wt % HF in an ice bath, and the mixture was stirred at 100 rpm for 96 h at room temperature. After the etching process, the sedimented V_4_C_3_T*_*x*_* was washed several times with DDW until pH ∼ 7. At this stage, we obtained multilayered (ML) V_4_C_3_T*_*x*_*. To further transform ML into delaminated V_4_C_3_T*_*x*_* nanosheets, we carried out a delamination process by using a tetramethylammonium hydroxide (TMAOH, 25 wt %, Sigma-Aldrich) solution in a ratio of 10 mL per 500 mg of MXene. The mixture was stirred for 24 h at room temperature. Next, the mixture was washed with DDW (10,000 RCF for 5 min) until the pH ∼ 7. Furthermore, the MXene was left to sediment for 1h, and the supernatant consisting of delaminated V_4_C_3_T*_*x*_* nanosheets was collected, while the sediment containing ML V_4_C_3_T*_*x*_* was discarded. In this work, the delaminated V_4_C_3_T*_*x*_* will be referred to as V_4_C_3_T*_*x*_* for simplicity.

### 2.4 Synthesis of Protonated Graphitic Carbon Nitride (PCN)

Graphitic carbon nitride (g-C_3_N_4_) is typically synthesized by heating 20 g of urea in air in a ceramic crucible covered with aluminum foil at 550 °C for 2 h. The heating rate was set at 10 °C/min, followed by natural cooling overnight. The obtained bulk g-C_3_N_4_ was further ground using a mortar to obtain fine powders. Next, 250 mg of bulk g-C_3_N_4_ was stirred in 5 mL of 12 M HCl for 1h at 300 rpm to obtain protonated g-C_3_N_4_ (PCN). Then, PCN was washed with DDW at 10,000 RCF for 5 min until the pH reached ∼7. To obtain a 2D nanosheet structure, we bath-sonicated PCN for 1 h. At the end, we separated PCN nanosheets by centrifugation at 1500 RCF for 10 min and collected the supernatant.

### 2.5 Preparation of V_4_C_3_T*_*x*_*/PCN Heterostructures

The V_4_C_3_T*_*x*_*/PCN heterostructures were formed by a facile electrostatic self-assembly method. Specifically, V_4_C_3_T*_*x*_* and PCN aqueous dispersions (each 2 mg/mL) were stirred together at 300 rpm for 1 h at room temperature. We varied the ratio of V_4_C_3_T_*x*_ to PCN components in the heterostructure, i.e., 1:3, 1:1, and 3:1 by mass.

### 2.6 Characterization

The scanning electron microscopy (SEM) and scanning transmission electron microscopy images (STEM) of the V_4_C_3_T*_*x*_*/PCN heterostructure were studied by field emission scanning microscopy (FE-SEM, Hitachi S5500, Hitachi, Tokyo, Japan). The images were acquired by mounting the samples on a carbon-coated copper grid (Ted Pella Inc., Redding, CA, USA). The same FE-SEM setup enables energy-dispersive X-ray spectroscopy (EDS). Raman spectra were recorded using an in situ Raman setup (ReactRaman, Mettler-Toledo, US), operating at 785 nm, a power of 400 mW, and an acquisition time of 30 s. The Raman spectra were obtained by measuring the samples in aqueous dispersion. X-ray diffraction patterns (XRD) were obtained from drop-cast samples using a BrukerD8 X-ray diffractometer. The zeta potential of PCN and V_4_C_3_T*_*x*_* was performed by a Zetasizer NANO ZS ZEN3500 and Advance Series – Lab (Blue Label), Malvern, respectively, using a plastic micro cuvette from Malvern Panalytical.

The chemical composition of the samples was studied by using X-ray photoelectron spectroscopy (XPS). XPS analyses were carried out using a Thermo Scientific KAlpha X-ray photoelectron spectrometer with an aluminum X-ray source (energy 1.4866 keV) and working at a pressure of 10^–8^–10^–9^ mbar in the main chamber. The X-ray spot size was settled at 400 μm. The samples were prepared by drop-casting onto conductive copper tape and dried under nitrogen (N_2_). All XPS spectra were calibrated by using the C_1s_ peak at 284.8 eV as a reference.

UV–visible (UV–vis) diffuse reflectance spectra of the samples were measured with a UV–vis spectrometer (Evolution 220, Thermo Scientific) equipped with an integrated sphere. The spectra were recorded with an integration time of 0.3 s, a wavelength resolution of 1 nm, and a scanning speed of 200 nm/min.

Evaluation of photoluminescence (PL) and time-resolved PL (TRPL) was done by employing a picosecond 375 nm laser diode, an objective microscope, a 405 nm upper pass filter, and a high-sensitivity visible light spectrometer. For TRPL measurements, we shifted the laser diode from continuous wave (CW) mode to pulsed mode at a 10 MHz repetition rate. The emitted PL signal after filtering was directed to a single-photon avalanche photodiode. The temporal characteristics of this signal were examined by using time-correlated single-photon counting electronics. Temperature-dependent PL and TRPL measurements were conducted using a Linkam HFS600E-PB4 cryostat with liquid nitrogen cooling, facilitating measurements from 70 to 370 K in 50K steps.

### 2.7 Optoelectronic Measurement

We investigated the optoelectronic properties of V_4_C_3_T*_*x*_*/PCN thin films by fabricating drop-cast thin films on glass substrates. The thin films were prepared as follows: first, glass substrates were immersed in Hellmanex cleaning concentrate (2 vol %) for 20 min, followed by rinsing with isopropanol and DDW. The substrates were then dried on a hot plate at 150 °C for 3 min. Before coating, the substrates were treated with UV–O_3_ for 30 min to promote surface hydrophilicity. Next, 150 μL of the V_4_C_3_T*_*x*_*/PCN aqueous solution (2 mg/mL) was drop-casted onto the substrates by a micropipette and allowed to dry overnight at room temperature. To improve the interfacial adhesion of the film and the substrate, the obtained coating was sintered at 110 °C for 1h. Here, V_4_C_3_T_*x*_ and V_4_C_3_T*_*x*_*/PCN heterostructured thin films were fabricated without polymeric binders. The edges of the thin films were cast with silver paste and spaced 1 cm apart, providing an electrical connection. Current–voltage curves were measured using a sourcemeter unit (Keithley SMU 2450, USA), within a measurement range of −5 to 5 V. For studying the photoresponsive properties of the films, the samples were placed in a photochemical LED reactor (Photocube Photochemical reactor, ThalesNano, Budapest, Hungary) and irradiated with white (400–700 nm, luminous flux 5920 lm) or UV light (365 nm, radiant flux of 44.8 W). Furthermore, the multicolor photocurrent density of the samples was measured using a digital multimeter (Keithley DAQ 6510, USA), with a bias voltage of 1.5 V and five alternating light on-and -off cycles (each cycle was carried out for 60 s).

### 2.8 Electrochemical Impedance Spectroscopy (EIS) Characterization

EIS characterizations were conducted in three-electrode systems using a Metrohm Dropsens potentiostat (μStat-i 400s), with 0.5 M Na_2_SO_4_ electrolyte. Ag/AgCl (in saturated KCl) and platinum wire were used as the reference and counter electrodes, respectively. Moreover, the previously prepared V_4_C_3_T*_*x*_*/PCN heterostructured thin films grown on ITO substrates were used as working electrodes via a drop-casting method similar to that explained before. EIS measurements were performed within the frequency range of 0.1–100 kHz with an AC voltage of 10 mV at the open-circuit potential in the dark condition.

## 3 Results and Discussion

**Figure 1:**
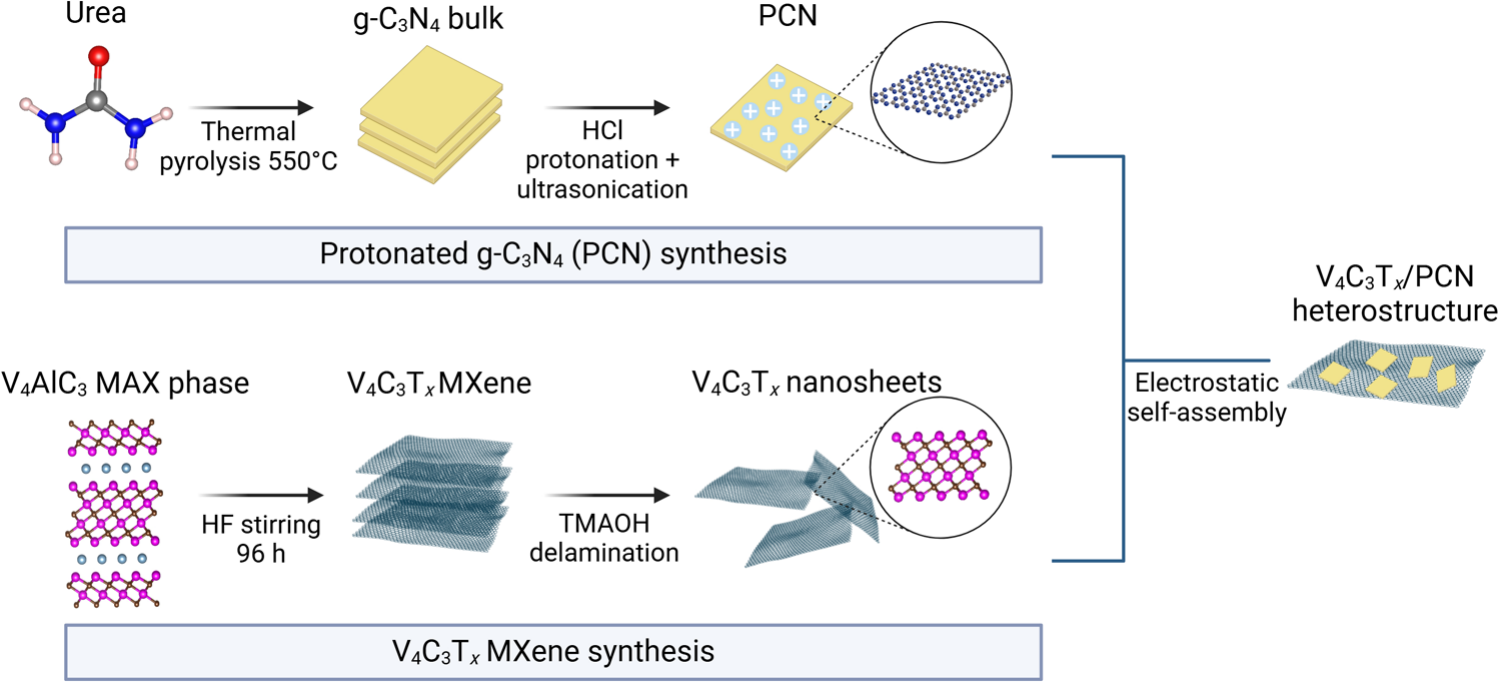
Schematic illustration of the V_4_C_3_T_*x*_/PCN synthesis.

**Figure 2:**
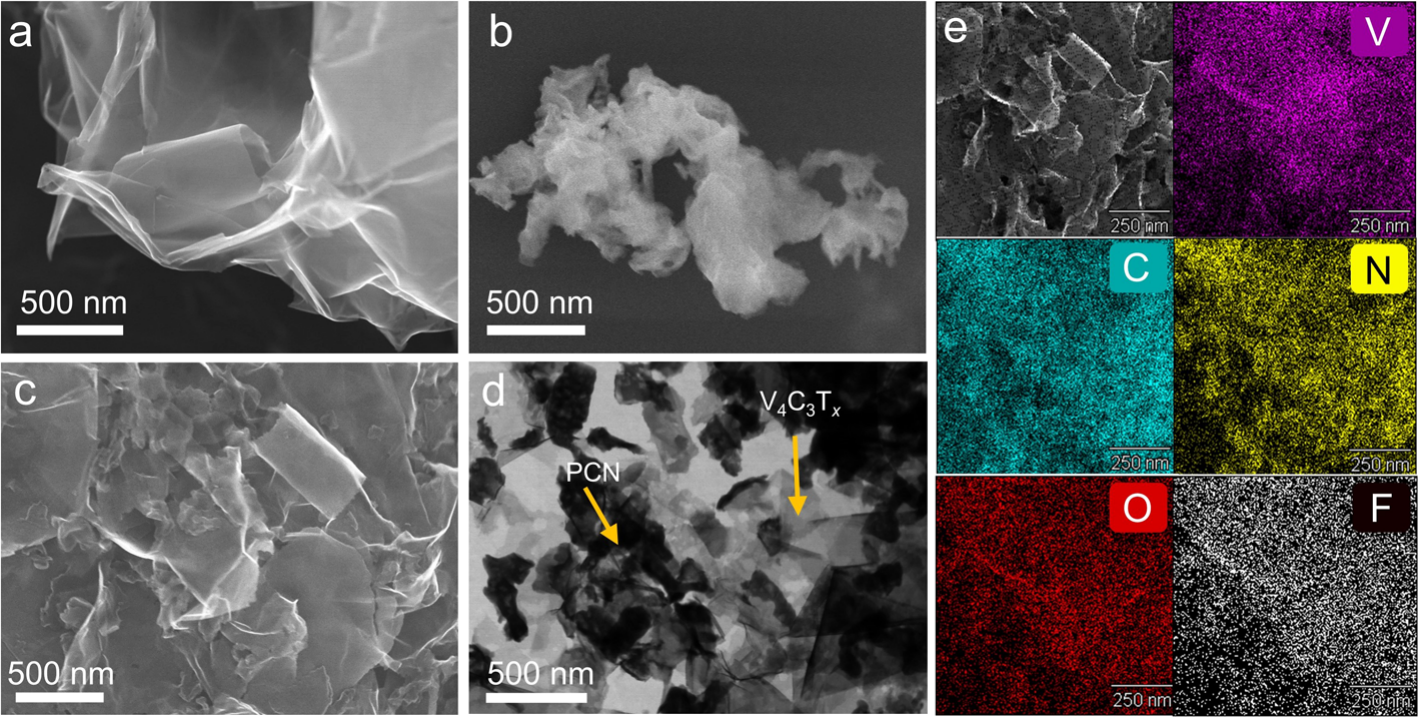
HR-SEM images of (a) V_4_C_3_T_*x*_, (b) protonated g-C_3_N_4_ (PCN), and (c) V_4_C_3_T_*x*_/PCN 1:1 heterostructure. (d) STEM images and (e) EDS elemental mapping of the V_4_C_3_T_*x*_/PCN 1:1 heterostructure.

The step-by-step synthesis of the V_4_C_3_T_*x*_/PCN heterostructure is illustrated in Figure 1. The ratio of V_4_C_3_T_x_ to PCN components in the heterostructure formulation was varied (i.e., 1:3, 1:1, and 3:1 by mass) to optimize the intrinsic optoelectronic properties. The starting V_4_AlC_3_ exhibits a bulk structure (Figure S1a). After 96 h of HF etching was performed, the accordion-like structure of multilayered (ML) V_4_C_3_T*_*x*_* was formed, as revealed in the SEM image (Figure S1b). After delamination, we obtained ultrathin V_4_C_3_T*_*x*_* nanosheets with smooth edges and wrinkled structures, as depicted in Figure 2a. The energy-dispersive X-ray spectroscopy (EDS) elemental analysis confirms the successful Al etching in V_4_C_3_T*_*x*_*, as evidenced by reduced Al to trace amounts (Figure S2). We observe the presence of V and C atoms, the main components in V_4_C_3_T*_*x*_*, as well as O and F, which are associated with the surface functional groups of MXene, such as −F, −OH, and −O.^22^ The second building block of the heterostructure, PCN, exhibits a crumpled nanosheet structure with a few hundred nanometer lateral sizes (Figure 2b). The thin-layer structure of both V_4_C_3_T*_*x*_* and PCN facilitates 2D/2D interfacial contacts, which may be advantageous for designing heterostructures with tight interfacial connections.^48^

The protonation of g-C_3_N_4_ into PCN can tune its surface charge from a negative value (−10.76 mV) to a positive zeta potential value (+6.92 mV) (Figure S3). Therefore, a highly negative surface charge of V_4_C_3_T*_*x*_* MXene (−44.36 mV) and a positive surface charge of PCN (+6.92 mV) facilitate the formation of V_4_C_3_T*_*x*_*/PCN heterostructures via electrostatic interactions. The SEM image of the 1:1 V_4_C_3_T*_*x*_*/PCN heterostructure shows a well-stacked 2D/2D nanosheet morphology (Figure 2c). We further confirm the interfacial connection between V_4_C_3_T*_*x*_* and PCN via scanning transmission electron microscopy (STEM). Here, we can observe the intimate interfacial contact between V_4_C_3_T*_*x*_* and PCN, where PCN with smaller nanosheet dimensions uniformly decorates the transparent V_4_C_3_T*_*x*_* nanosheets (Figure 2d). Well-defined EDS elemental mapping of V_4_C_3_T*_*x*_*/PCN 1:1 further confirms that PCN successfully decorated the V_4_C_3_T*_*x*_* nanosheets, as evidenced by the homogeneous distribution of V, C, N, O, and F elements (Figure 2e).

**Figure 3:**
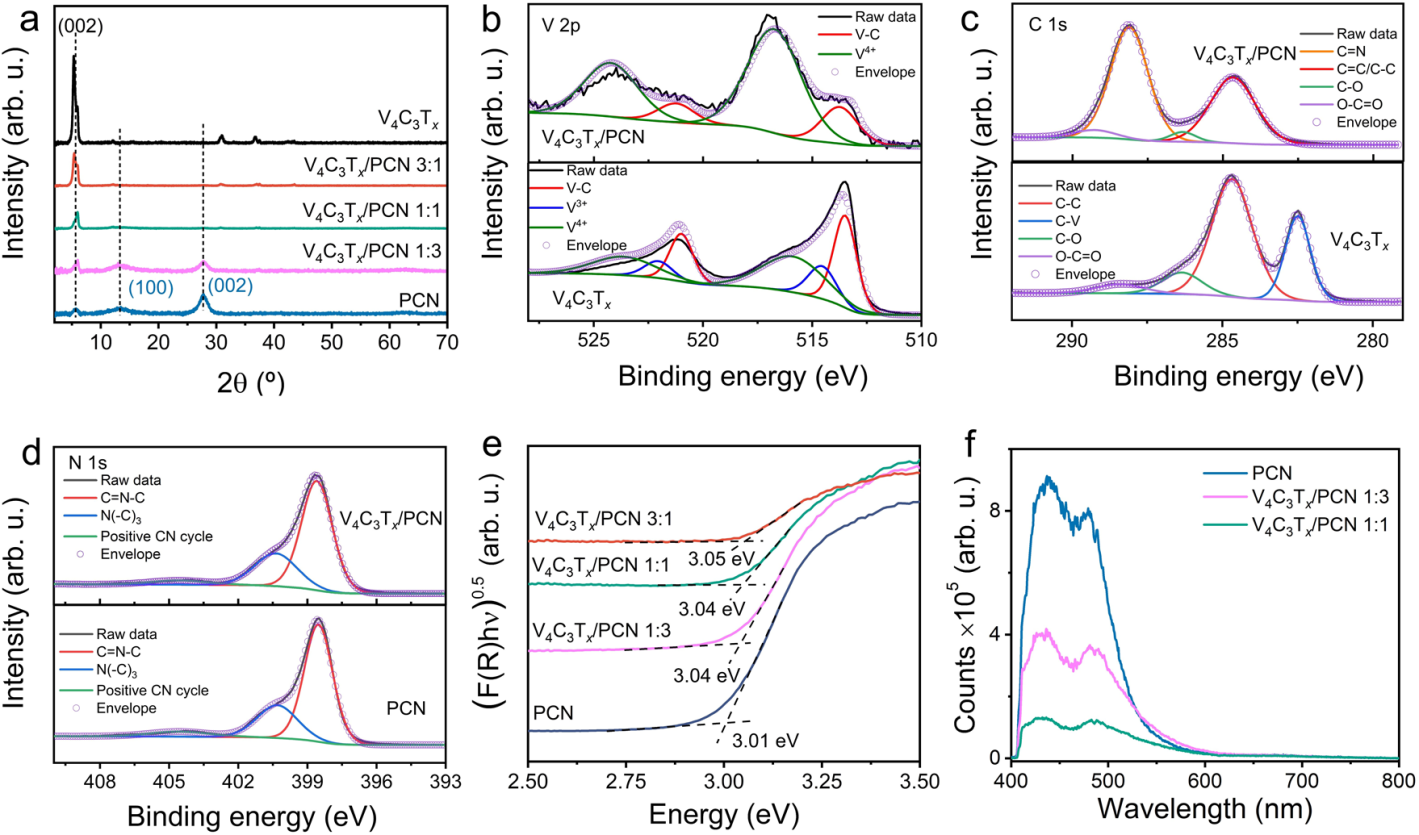
(a) XRD of V_4_C_3_T_*x*_, PCN, and V_4_C_3_T_*x*_/PCN heterostructures. High-resolution XPS spectra of (b) V 2p, (c) C 1s, and (d) N 1s for V_4_C_3_T_*x*_, PCN, and V_4_C_3_T_*x*_/PCN 1:1. (e) Tauc plots of PCN and V_4_C_3_T_*x*_/PCN heterostructures. (f) Steady-state photoluminescence spectra of PCN and V_4_C_3_T_*x*_/PCN heterostructures at 270 K.

The crystal structure of the synthesized samples was analyzed via X-ray diffraction (XRD). The precursor V_4_AlC_3_ MAX phase shows an intense peak of (0010) located at 39.75°, which is in good agreement with the literature.^28^ After performing etching and delamination, we successfully transformed V_4_AlC_3_ into V_4_C_3_T*_*x*_* MXene, as evidenced by the absence of the (0010) peak of the MAX phase and the broadening and shifting of the (002) peak to lower energy (from 7.80° to 5.29°) (Figure S4). This shifting indicates a larger interlayer spacing in V_4_C_3_T_*x*_ MXene compared to V_4_AlC_3_ due to the removal of the Al layer.^28,29^ The XRD pattern of PCN shows typical (100) and (002) peaks, which can be assigned to the intraplanar structural packing of s-triazine units and interlayer π–π stacking of g-C_3_N_4_, respectively.^30^ Compared to bulk g-C_3_N_4_, PCN shows a weakening of the (100) peak, indicating a decrease in the planar size of polymeric *s*-triazine units (Figure S5).^31^ Interestingly, the overall crystal structure of PCN is still maintained, suggesting that the protonation treatment does not compromise the structural integrity of g-C_3_N_4_.^32^ Importantly, the XRD patterns of V_4_C_3_T_*x*_/PCN heterostructures show the coexistence of each heterostructure components, i.e., the (002) peak of V_4_C_3_T_*x*_ and the (100) and (002) peaks of PCN (Figure 3a).

Raman spectra further support the successful formation of V_4_C_3_T*_*x*_*/PCN heterostructures (Figure S6). As the PCN content increases, the baseline of the Raman spectra rises, attributed to the fluorescence effect of PCN.^33^ Moreover, after PCN hybridization with V_4_C_3_T*_*x*_*, we observe several Raman-active peaks. The peaks located at 691 cm^–1^ can be assigned to the C–N ring breathing modes of s-triazine.^34^ The peaks in the range of 1200–1700 cm^–1^ originate from C–N stretching vibrations, resembling typical D- and G-bands of graphitic carbon-based materials.^35^

X-ray photoelectron spectroscopy (XPS) was used to investigate the surface chemistry and chemical composition of the samples. Figure S7 shows the XPS survey spectra of V_4_C_3_T*_*x*_*, PCN, and V_4_C_3_T*_*x*_*/PCN heterostructures. Notably, we observe the peaks of V 2p, C 1s, F 1s, and O 1s in the XPS survey spectra of V_4_C_3_T*_*x*_*. The atomic concentration determined from XPS measurements is given in Table S1. The absence of peaks corresponding to Al and other impurities confirms the high purity of the synthesized MXene. After the hybridization of V_4_C_3_T_*x*_ with PCN, a new peak corresponding to N 1s emerges in the XPS survey spectra, while the intensity of the V 2p peak weakens. This result confirms the uniform surface coverage of V_4_C_3_T*_*x*_* nanosheets by PCN, as observed in SEM images.

The high-resolution V 2p XPS spectra of V_4_C_3_T*_*x*_* can be fitted by three doublet components of V 2p_3/2_ and V 2p_1/2_ (Figure 3b).^22,27^ The peaks at 513.5 and 521.0 eV can be attributed to the V–C (V^2+^),^36,37^ while those at 514.6 and 522.1 eV can be attributed to V^3+^. The highest binding energy peaks, i.e., 516.1 and 523.7 eV, are assigned to V^4+^. Here, V^3+^ and V^4+^ may arise from the surface oxidation of V_4_C_3_T*_*x*_*.^27^ For the V_4_C_3_T*_*x*_*/PCN heterostructure, the V–C peak shifts slightly to 513.7 and 521.2 eV, and the V^3+^ peak is not observed. In contrast, the V^4+^ peak is enhanced and shifts to higher binding energies (516.7 and 524.3 eV). These shifts imply strong interfacial interactions and charge transfer between V_4_C_3_T*_*x*_* and PCN, with electrons transferring from V_4_C_3_T_*x*_ to PCN.^38,39^

Figure 3c shows the C 1s XPS spectra of the V_4_C_3_T*_*x*_* and V_4_C_3_T*_*x*_*/PCN heterostructures. Four components fit the spectra of V_4_C_3_T*_*x*_* C 1s. The peak at 282.5 eV can be attributed to the C–V bond of V_4_C_3_T*_*x*_*.^27^ The peaks located at 284.7, 286.3, and 288.4 eV can be assigned to C–C/C=C, C–O, and O–C=O, respectively.^22^ In the case of V_4_C_3_T*_*x*_*/PCN, a new intense peak located at 288.1 eV can be observed, which can be assigned to sp^2^-hybridized C bonded to N in triazine rings (C=N), as can also be seen in C 1s of PCN (Figure S8).^40^ The C–V component cannot be clearly identified in the V_4_C_3_T*_*x*_*/PCN heterostructure due to the surface coverage of V_4_C_3_T*_*x*_* by PCN. Additionally, a carbon atom is located at the inner layer compared to a vanadium atom in the V_4_C_3_T*_*x*_* layered structure. The N 1s spectrum of PCN can be fitted with three components (Figure 3d). The peaks located at 398.6 and 400.3 eV can be assigned to C–N=C and N–(C)_3_ groups, respectively.^32^ Furthermore, the peak at 404.5 eV appears because of the successful protonation treatment, which makes the CN heterocycles positively charged, consistent with the zeta potential results.^32^ In the V_4_C_3_T*_*x*_*/PCN heterostructure, we observe a similar N 1s spectrum in agreement with PCN. Therefore, XRD, Raman, and XPS results confirm the successful formation of the 2D/2D heterostructure via the electrostatic self-assembly method.^20,41^

We then conducted optoelectrical characterizations of the as-synthesized V_4_C_3_T*_*x*_*/PCN heterostructures. Examining these intrinsic properties is crucial for extending our understanding of g-C_3_N_4_-based heterostructures beyond photocatalysis applications. First, we examined the optical absorption of V_4_C_3_T*_*x*_*, PCN, and V_4_C_3_T*_*x*_*/PCN heterostructures via ultraviolet–visible (UV–vis) diffuse reflectance spectra (DRS) measurements; the measurements were taken in thin film form (Figure S9). V_4_C_3_T*_*x*_* exhibits a strong broadband absorption in the measured wavelength range (300–1000 nm), spanning from UV to near-infrared regions (NIR). The absence of an absorption edge is related to its metallic band structure.^23^ For PCN, we observe a distinct absorption edge at ∼420 nm, related to the excitonic band-to-band electronic transition of typical semiconducting materials. This value is in agreement with a few-layer g-C_3_N_4_ nanosheet reported by Jiang et al.^30^ We observe a similar absorption edge feature in all of the V_4_C_3_T*_*x*_*/PCN heterostructures. Moreover, increasing the V_4_C_3_T*_*x*_* content also increases the optical absorption of the heterostructure in the visible region. Therefore, using MXene into the PCN heterostructure is beneficial for improving its light absorption performance.^48^ Interestingly, V_4_C_3_T*_*x*_*/PCN heterostructures can be used as soluble ink for postprocessing (Figure S10). Figure S11 shows the drop-casted thin films with color variations, where the V_4_C_3_T*_*x*_*/PCN films get darker by adding more V_4_C_3_T*_*x*_*, implying stronger light absorption properties. It should be noted that PCN exhibits an opaque white color, which differs from the well-known bulk g-C_3_N_4_ with a typical yellow color, which can be attributed to the transformation from bulk to few-layered nanosheets.^42^

We used the Kubelka–Munk method to determine the optical bandgap values of PCN and V_4_C_3_T*_*x*_*/PCN heterostructures (Figure 3e). The extracted bandgap of PCN is 3.01 eV, larger than the reported bulk g-C_3_N_4_ with a typical bandgap of 2.73 eV. However, the value is similar to 2D g-C_3_N_4_ formed by tri*s*-triazine blocks.^43^ The plausible reason for the obtained bandgap larger than the bulk counterpart is related to the strong quantum confinement effect in PCN nanosheets, shifting the conduction and valence bands to opposite directions.^30^ The band gap of V_4_C_3_T*_*x*_*/PCN shows a negligible change compared to pure PCN. This result suggests that the intrinsic bandgap of the heterostructure is attributed mainly to the electronic transition from the valence band to the conduction band of semiconducting PCN, without the introduction of new energy levels induced by V_4_C_3_T*_*x*_* hybridization.^48^

To study the electronic structure and charge transfer of photogenerated charge carriers in V_4_C_3_T*_*x*_*/PCN heterostructures, we performed temperature-dependent photoluminescence (PL) and time-resolved photoluminescence (TRPL) measurements. The steady-state PL spectrum of PCN shows broad visible luminescence in the range of 400–600 nm with observed peaks at 430 and 485 nm (Figure 3f). In PCN, the valence band is formed by the σ bond of C–N with sp^3^ hybridization, the π bond of C–N with sp^2^ hybridization, and N_2_ lone pairs (LP). Meanwhile, the conduction band is the superposition of the excited σ and π bonds (σ* and π*).^44^ To resolve the origin of the electronic transition in PCN, we performed Gaussian fitting (Figure S12). Here, the broad PL peak of PCN at 485 nm can be fitted by two components at 472 and 521 nm. Notably, the peak at 430 nm can be assigned to the transition from the σ* conduction band to the LP valence band. The second peak at 472 nm originated from the transition of the σ* conduction band to the π valence band. The shoulder peak at 521 nm is attributed to the transition of π* conduction band to the LP valence band.^45,46^ We further compare the PL spectra of PCN and V_4_C_3_T*_*x*_*/PCN heterostructures at a temperature of 270 K. It is important to note that heterostructure formation may improve photogenerated charge transfers and exciton splitting, decreasing PL emission.^47^ In the present case, the PL spectra of V_4_C_3_T*_*x*_*/PCN heterostructures are quenched compared to pure PCN, where 1:1 V_4_C_3_T*_*x*_*/PCN exhibits the weakest luminescence, suggesting PL quenching and efficient charge transfer across the 2D/2D interface, as supported by XPS results. Moreover, the PL emission of V_4_C_3_T*_*x*_*/PCN 3:1 cannot be detected, which might be attributed to light-shielding effects from the excessive amount of metallic V_4_C_3_T*_*x*_* in the heterostructure formulation.^5,48^ Thus, it is important to control the ratio between V_4_C_3_T*_*x*_* and PCN to ensure that light can be absorbed by the photoactive material, i.e., PCN, in order to form excitons.

We then studied the temperature-dependent PL spectra of the samples to analyze the photogenerated charge carrier dynamics. To the best of our knowledge, this is the first study exploring the photophysics of g-C_3_N_4_-based heterostructures *via* a combination of temperature-dependent PL and TRPL. Figure S13 shows the temperature-dependent PL spectra (70–370 K) of PCN, V_4_C_3_T*_*x*_*/PCN 1:3, and V_4_C_3_T*_*x*_*/PCN 1:1. In all samples, we observe a major peak located at 430 nm, an asymmetrical peak at 485 nm, and a broad peak located at 700 nm. Interestingly, the peak at 700 nm gets stronger by increasing V_4_C_3_T*_*x*_* amount in V_4_C_3_T*_*x*_*/PCN hybrids and can only be observed at low-temperature measurements (below 120 K). While we have discussed the origin of the PL peaks at 430 and 485 nm, the occurrence of the PL peak at 700 nm might be related to either radiative recombination of excitons bound to defects or hybridized states at the heterostructure interface. Furthermore, we observed that the emission centered at 485 nm dominated over the 430 nm emission by increasing the measurement temperature. One of the plausible reasons is the nonradiative defect electron trapping process, resulting in exciton quenching.^49^ Next, we evaluated the stability of the 430 nm PL signal at room temperature by performing repeated heat treatments at 370 K (Figure S14). The PL signal is observable after performing heat treatment, indicating the decent thermal stability of the V_4_C_3_T*_*x*_*/PCN heterostructure. It is known that V_4_C_3_T*_*x*_* exhibits high thermal stability compared to other MXenes.^24^

**Figure 4:**
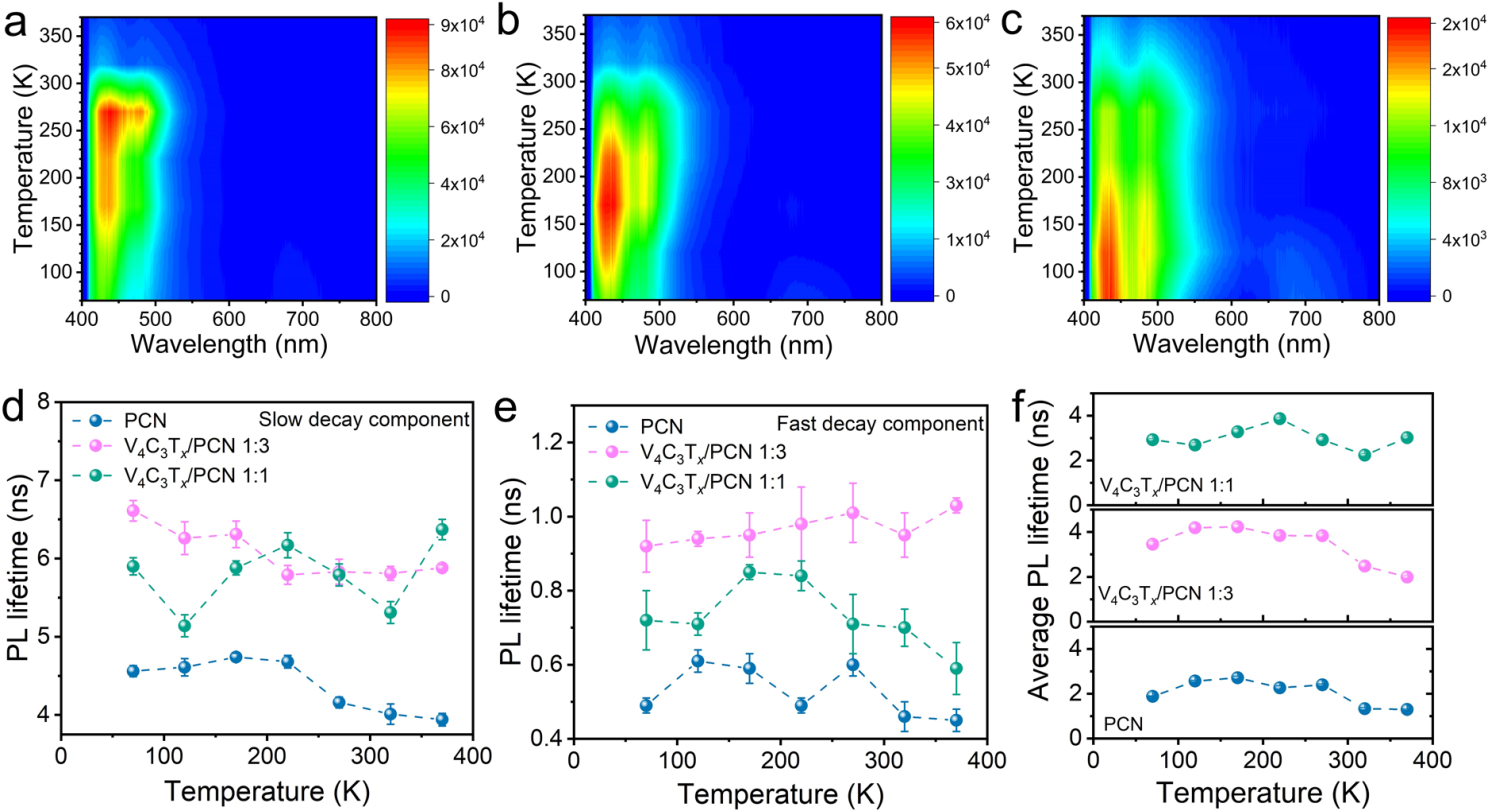
PL contour maps at various temperatures from 70 to 370 K for (a) PCN, (b) V_4_C_3_T_*x*_/PCN 1:3, and (c) V_4_C_3_T_*x*_/PCN 1:1. PL lifetime of the (d) slow decay component, (e) fast decay component, and (f) average for PCN, V_4_C_3_T_*x*_/PCN 1:3, and V_4_C_3_T_*x*_/PCN 1:1 as a function of the temperature.

Interestingly, PCN shows noticeable negative thermal quenching (NTQ), where PL intensity increases in the temperature range of 70–270 K (Figure 4a), followed by a decrease in intensity due to typical thermal quenching above 270 K. In the case of V_4_C_3_T_*x*_/PCN 1:3, both NTQ and typical thermal quenching are observed (Figure 4b). Similar temperature-dependent luminescence behavior is also observed in halide perovskite crystals and II–VI semiconductors.^50−52^ The presence of NTQ can be related to defects or shallow trap states located near the band edge, acting as a barrier for carrier trapping.^53^ Godin et al. observed a significant density of trap states in g-C_3_N_4_ located at 1 eV below the band edges.^54^ Furthermore, PL contour maps of V_4_C_3_T*_*x*_*/PCN 1:1 (Figure 4c) exhibit different features, where only typical thermal quenching is observed without NTQ. We also observed that the peak located at 700 nm became more pronounced compared to PCN and V_4_C_3_T*_*x*_*/PCN 1:3 at the measurement temperature below 120 K. This distinct feature implies that the addition of V_4_C_3_T*_*x*_* in the V_4_C_3_T*_*x*_*/PCN 1:1 formulation promotes a different luminescence mechanism. Furthermore, the thermal quenching fitting results and the extracted parameters of PCN and V_4_C_3_T*_*x*_*/PCN heterostructures are given in Figure S15 and Table S2, respectively. Interestingly, V_4_C_3_T*_*x*_*/PCN 1:1 shows an activation energy of 139.5 meV, higher than the previously reported activation energy of g-C_3_N_4_ (73.58 meV).^46^

Temperature-dependent TRPL decay curves of PCN and V_4_C_3_T*_*x*_*/PCN heterostructures show similar behavior that can be fitted with a double exponential function (Figure S16). The extracted fitting parameters, i.e., weight contribution and lifetime, are presented in Tables S3–S5. The fast decay component (τ_1_) is related to direct exciton recombination, whereas the slow decay component (τ_2_) is originated from the recombination of electrons trapped by defects.^55^Figure 4d shows the PL lifetime of the slow decay component for PCN and V_4_C_3_T*_*x*_*/PCN heterostructures. Notably, V_4_C_3_T*_*x*_*/PCN 1:1 and V_4_C_3_T*_*x*_*/PCN 1:3 show prolonged lifetimes compared to PCN at all measured temperatures. The same trend is observed in the fast decay component (Figure 4e). Based on the fitting parameters, besides the prolonged PL lifetime, the hybridization of PCN by V_4_C_3_T*_*x*_* caused the contribution of the slow decay component to become more prominent, implying that the emission mechanism can be influenced by the occurrence of trap states, promoting long interfacial charge separation times.^52,56^

To facilitate easier comparison, we plot the average PL lifetime of PCN, V_4_C_3_T*_*x*_*/PCN 1:3, and V_4_C_3_T*_*x*_*/PCN 1:1 in one frame, as depicted in Figure 4f. The average PL lifetime of PCN within the temperature range of 70–370 K ranges at 1.88–2.71 ns. The short photogenerated charge carrier lifetime is a well-known bottleneck in developing g-C_3_N_4_ as optoelectronic devices and photocatalysts.^57^ The PL lifetime is prolonged in V_4_C_3_T*_*x*_*/PCN heterostructures, with a magnitude of 1.99–4.19 and 2.24–3.86 ns for V_4_C_3_T*_*x*_*/PCN 1:3 and 1:1, respectively, at 70–370 K. This improvement can be connected to the efficient interfacial charge transfer at the 2D/2D interface of PCN and V_4_C_3_T*_*x*_*, inducing electrons to travel longer to recombine, subsequently getting electrons trapped into defect states.^58^ Interestingly, the PL lifetime of V_4_C_3_T*_*x*_*/PCN 1:1 remains stable at all evaluated temperatures, indicating this sample’s good stability and the potential to be used as a building block of optoelectronic devices operating at elevated temperatures (e.g., ∼370 K).^59^ Here, the optimum ratio between PCN and V_4_C_3_T*_*x*_*, i.e., 1:1, creates new emission pathways, i.e., interfacial charge transfers, resulting in a stable PL lifetime across various temperatures (i.e., 70–370 K). However, in the case of PCN and V_4_C_3_T*_*x*_*/PCN 1:3, the lifetime decreases by increasing the temperature as PL relaxation dynamics become faster.^45^ Whereas, in the case of V_4_C_3_T*_*x*_*/PCN 3:1, the excess amount of V_4_C_3_T*_*x*_* leads to light-shielding effects, which is not beneficial for light-emitting applications. By comparing the PL lifetime at the elevated temperature (370 K), V_4_C_3_T*_*x*_*/PCN 1:1 exhibits a 2.3-fold longer lifetime than pristine PCN. Based on PL and TRPL analyses, V_4_C_3_T*_*x*_*/PCN 1:1 shows interesting intrinsic optoelectronic properties, benefiting from the synergistic combination of V_4_C_3_T*_*x*_* and PCN.

**Figure 5:**
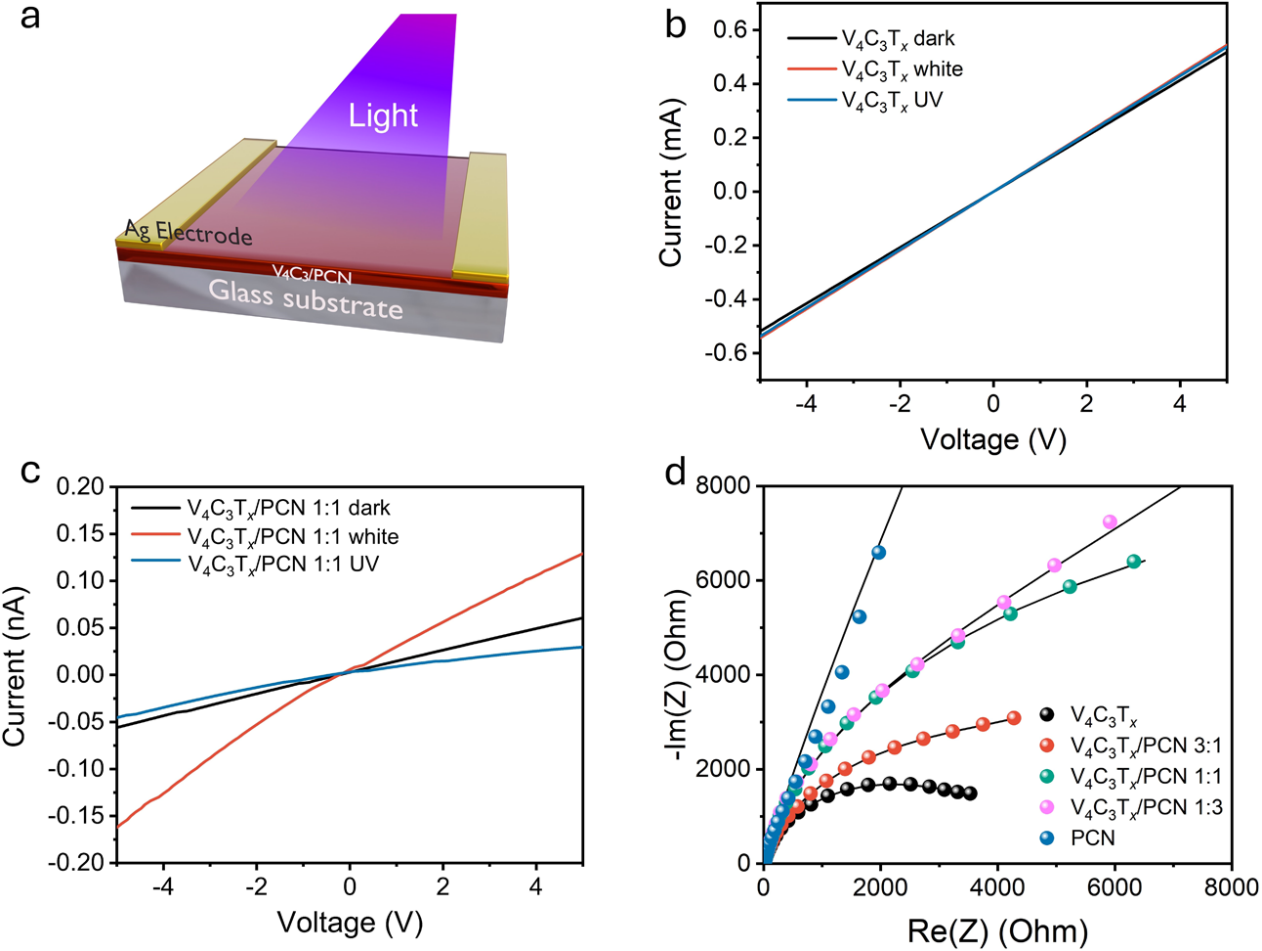
(a) Measurement architecture used to assess the photosensitivity of the as-prepared samples. Current–voltage curves in the dark and under white or UV light irradiation (wavelength 365 nm) of (b) V_4_C_3_T_*x*_ and (c) V_4_C_3_T_*x*_/PCN 1:1. (d) Nyquist plot of the V_4_C_3_T_*x*_, PCN, and V_4_C_3_T_*x*_/PCN heterostructures.

The photosensitivity of V_4_C_3_T*_*x*_*/PCN heterostructures was evaluated using a straightforward metal–semiconductor-metal device, fabricated by drop-casting V_4_C_3_T*_*x*_*/PCN aqueous ink onto a glass substrate. This thin film was prepared without the need for an additional polymeric binder. Figure 5a shows the architecture employed to measure the optoelectronic properties of the samples. The samples were exposed to UV or white light, and their corresponding current–voltage (*I*–*V*) curves were recorded. V_4_C_3_T*_*x*_* exhibits an ideal ohmic contact with no photosensitivity toward UV and white light, as evidenced by negligible current changes in Figure 5b. This behavior is expected because nonoxidized V_4_C_3_T*_*x*_* MXene acts as a metallic material,^23^ resulting in limited light–matter interactions for effective light-harvesting.^5,17^ To investigate the effects of oxidation on the optoelectronic properties, a 9-month-aged V_4_C_3_T*_*x*_* sample was tested. Figure S17 shows the UV–vis transmittance evolution of V_4_C_3_T*_*x*_* after aging, with an increase in the 400–800 nm region indicating oxidation. Simultaneously, a decrease in the UV-region peak suggests the presence of vanadium oxide species. Subsequently, the photoactivity of the aged V_4_C_3_T*_*x*_* was tested to assess the role of surface oxides. The aged samples demonstrated a lower dark current compared to that of fresh V_4_C_3_T*_*x*_*, implying degradation due to oxidation into vanadium oxide species. However, the oxide species contributed minimally to the photoresponse under UV and white light irradiation, as shown in Figure S18. Therefore, the superficial oxide had only a minor impact on the light absorption of V_4_C_3_T*_*x*_*.

Therefore, we expect that upon the hybridization of V_4_C_3_T*_*x*_* with PCN, the light–matter interaction in V_4_C_3_T*_*x*_*/PCN heterostructures improves. PCN (Figure S19a) and V_4_C_3_T*_*x*_*/PCN 1:3 (Figure S19b) exhibit poor electrical conductivity, exceeding the lowest measurement limit of the sourcemeter. In the case of V_4_C_3_T*_*x*_*/PCN 3:1, the current slightly increases with UV and white light irradiation (Figure S19c). However, the photocurrent enhancement is not strong enough, which can be caused by the light-shielding effects of excessive V_4_C_3_T*_*x*_* content, as we discussed before. Interestingly, high photosensitivity with a linear curve is observed in 1:1 V_4_C_3_T*_*x*_*/PCN, as can be seen in Figure 5c. Notably, V_4_C_3_T*_*x*_*/PCN 1:1 shows photosensitivity toward white light as well as UV light, which is beneficial for practical purposes as a large fraction of sunlight lies within the visible light region.^60^ A high photocurrent in 1:1 V_4_C_3_T*_*x*_*/PCN indicates a low probability of charge recombination and low charge transfer resistance across the interfaces, which are in line with PL and TRPL findings. Therefore, V_4_C_3_T*_*x*_*/PCN 1:1 is the optimum formulation to get sufficient electrical conductivity coupled to good photosensitivity.

We further evaluated the photocurrent of V_4_C_3_T*_*x*_*/PCN with various excitation wavelengths, spanning from red (625 nm) to UV light (365 nm). A high photocurrent is observed when the wavelengths reach blue light (457 nm), whereas a low photocurrent is acquired when the wavelengths are below this threshold (Figure S20). This photocurrent response agrees well with the optoelectronic characterization of V_4_C_3_T*_*x*_*/PCN, where the semiconducting PCN exhibits an absorption edge around this region. Electrochemical impedance spectroscopy (EIS) through the Nyquist plot was used to study the charge transfer resistance of V_4_C_3_T*_*x*_*, PCN, and V_4_C_3_T*_*x*_*/PCN heterostructures. The experimental results are well-fitted with the equivalent circuit model in Figure S21. In detail, PCN shows a high charge transfer resistance (*R*_CT_) with a magnitude of 27.58 kΩ (Table S6). The value of *R*_CT_ is significantly lower in the heterostructures, reaching *R*_CT_ of 14.50 kΩ (V_4_C_3_T*_*x*_*/PCN 1:3), 11.02 kΩ (V_4_C_3_T*_*x*_*/PCN 1:1), and 4.88 kΩ (V_4_C_3_T*_*x*_*/PCN 3:1) (Figure 5d). Here, V_4_C_3_T*_*x*_* lowers the resistance and promotes high electron mobility in the heterostructures, as V_4_C_3_T*_*x*_* has the smallest semicircle among all samples, indicating a low *R*_CT_. However, it is important to note that in the case of excess V_4_C_3_T*_*x*_* (V_4_C_3_T*_*x*_*/PCN 3:1), the *R*_CT_ is close to the pure V_4_C_3_T_*x*_, due to a strong metallic characteristic, which explains why we did not observe the luminescence in this sample. Therefore, we showcase that 1:1 V_4_C_3_T*_*x*_*/PCN became the optimized sample, showing excellent photoactivity and luminescence properties. Our results show that V_4_C_3_T*_*x*_*/PCN heterostructures can effectively promote efficient charge transfer across the interfaces and prolong photogenerated charge carrier lifetimes, which is beneficial to fabricate high-performing optoelectronic devices.

## 4 Conclusions

In conclusion, we have fabricated the 2D/2D V_4_C_3_T*_*x*_*/PCN heterostructure via an electrostatic self-assembly technique and studied its photophysics and intrinsic optoelectronic properties. The effect of V_4_C_3_T*_*x*_* addition to tune the optical properties of PCN was studied comprehensively by temperature-dependent PL and TRPL. The optimized sample (V_4_C_3_T*_*x*_*/PCN 1:1) showed efficient charge transfer across the 2D/2D interface, as demonstrated by PL quenching. We also observed a prolonged PL lifetime compared to pristine PCN at 70–370 K, with a 2.3-fold longer lifetime at elevated temperatures (370 K). Additionally, we observed high photosensitivity of V_4_C_3_T*_*x*_*/PCN 1:1 toward white and UV light, which is beneficial for harvesting a large portion of solar energy. The ability to achieve photosensitivity to both UV and white light without the need for additional polymeric binders is particularly encouraging and could be further refined through interfacial engineering. In the case of V_4_C_3_T*_*x*_*/PCN 1:3, we observed low electrical conductivity and photosensitivity toward light, indicating low photoactivity. The observed absence of light-emitting properties in the V_4_C_3_T*_*x*_*/PCN 3:1 configuration, attributed to light-shielding effects, further underscores the importance of optimizing material composition. The fabricated V_4_C_3_T*_*x*_*/PCN heterostructures open the potential for usage in advanced optoelectronic devices such as photodetectors, solar cells, solar batteries, and light-emitting devices. By addressing interfacial effects, such as structural defects, facet mismatching, and charge polarization, the performance of these devices can be substantially enhanced.

## Supporting Information Available

SEM images and EDS spectra of V_4_AlC_3_ MAX phase; zeta potential of bulk g-C_3_N_4_, PCN, and V_4_C_3_T*_*x*_* aqueous dispersion; XRD patterns of V_4_AlC_3_, V_4_C_3_T*_*x*_*, bulk g-C_3_N_4_, and PCN; absorption spectra and digital photograph of V_4_C_3_T*_*x*_*, PCN, and V_4_C_3_T*_*x*_*/PCN heterostructures; temperature-dependent PL and time-resolved PL spectra of V_4_C_3_T*_*x*_*, PCN, and V_4_C_3_T*_*x*_*/PCN heterostructures; additional current–voltage curve data of V_4_C_3_T*_*x*_*, PCN, and V_4_C_3_T*_*x*_*/PCN heterostructures; equivalent circuit model used for fitting the Nyquist plot; atomic concentrations of V_4_C_3_T*_*x*_*, PCN, and V_4_C_3_/PCN heterostructures obtained from XPS; fitting parameters of thermal quenching by integrating excitonic peak; extracted fitting parameters of temperature-dependent TRPL; and equivalent circuit parameters obtained from EIS fitting (PDF)
