# Supplementary material for: Unraveling the Role of Interfacial Charge Transfer on Photoactivity and Anomalous Luminescence Quenching of V4C3Tx/Protonated g-C3N4 Heterostructures
Source: ACS Appl Mater Interfaces. 2025 Mar 10;17(11):17454–64. doi: 10.1021/acsami.4c19729 (PMC11931479; doi:10.1021/acsami.4c19729)
Supplement: Supplementary file 1 — am4c19729_si_001.pdf [file am4c19729_si_001.pdf]

Supporting Information

Unraveling the Role of Interfacial Charge Transfer on Photoactivity and Anomalous Luminescence Quenching of $V_4C_3T_x$/Protonated g-C_3N_4 Heterostructures

Muhammad Abiyyu Kenichi Purbayanto[1,*] Madhurya Chandel[1], Michał Makowski[2], Muhammad Danang Birowosuto[2], Verónica Montes-García[3], Kaitlyn Prenger[5], Artur Ciesielski[3,4], Michael Naguib[5], Agnieszka Maria Jastrzębska[1,*]

[1]Warsaw University of Technology, Faculty of Mechatronics, św. Andrzeja Boboli 8, 02-525 Warsaw, Poland

[2]Łukasiewicz Research Network—PORT Polish Center for Technology Development, Stabłowicka 147, 54-066 Wrocław, Poland

[3]Université de Strasbourg, CNRS, ISIS 8 allée Gaspard Monge, 67000 Strasbourg, France

[4]Centre for Advanced Technologies, Adam Mickiewicz University, Uniwersytetu Poznańskiego 10, 61-614 Poznań, Poland

[5]Department of Physics and Engineering Physics, Tulane University, New Orleans, Louisiana 70118, United States

*email: muhammad_abiyyu.kenichi.dokt@pw.edu.pl, agnieszka.jastrzebska@pw.edu.pl

Supplementary methods

Study on the reversibility of photoluminescence after heat treatment

To investigate the reversibility of the photoluminescence (PL) signal after heat treatment, $V_4C_3T_x$/PCN 1:1 film was heated at the hot plate at 370 K for different time intervals. The PL signal was then measured at room temperature. Here, the evolution of the PL peak at 430 nm was traced to study the PL signal stability.

Fitting temperature-dependent photoluminescence

The integrated PL intensity of PCN and $V_4C_3T_x$/PCN 1:3 were fitted by an analytical model developed by Shibata et al.[1] which can be expressed as:

$$I = I_0 \frac{1 + A'.\exp\left(-\frac{E'}{k_b T}\right)}{1 + A.\exp\left(-\frac{E}{k_b T}\right)}$$

Where, I is the integrated PL intensity at absolute temperature T, I_0 is the maximum integrated intensity. A' and A is the negative thermal quenching coefficient and typical thermal quenching coefficient related to non-radiative electron excitation. E' and E are the activation energies for negative thermal quenching and typical thermal quenching.

Supplementary Figures

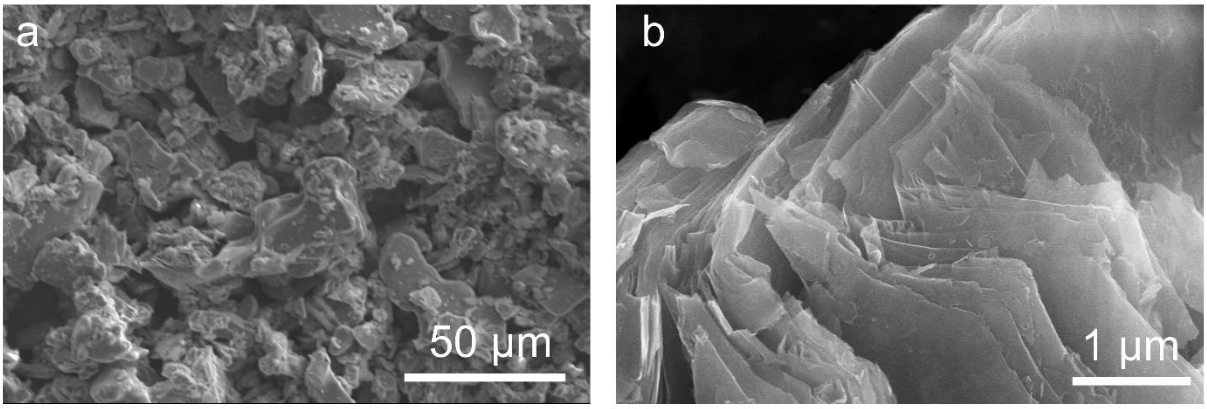

Figure S1. SEM images of (a) V_4AlC_3 MAX and (b) multilayered $V_4C_3T_x$ MXene.

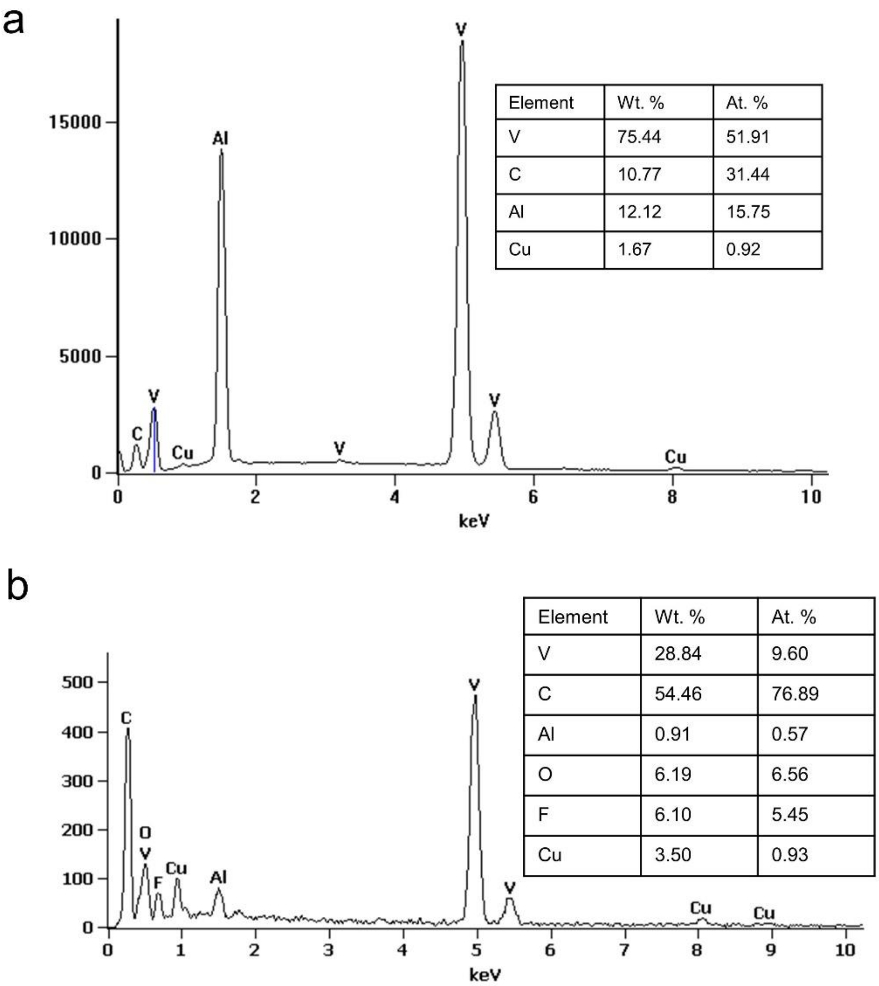

Figure S2. EDS spectra of (a) V_4AlC_3 MAX phase and (b) $V_4C_3T_x$. Excessive C contributions come from carbon-coated copper grid.

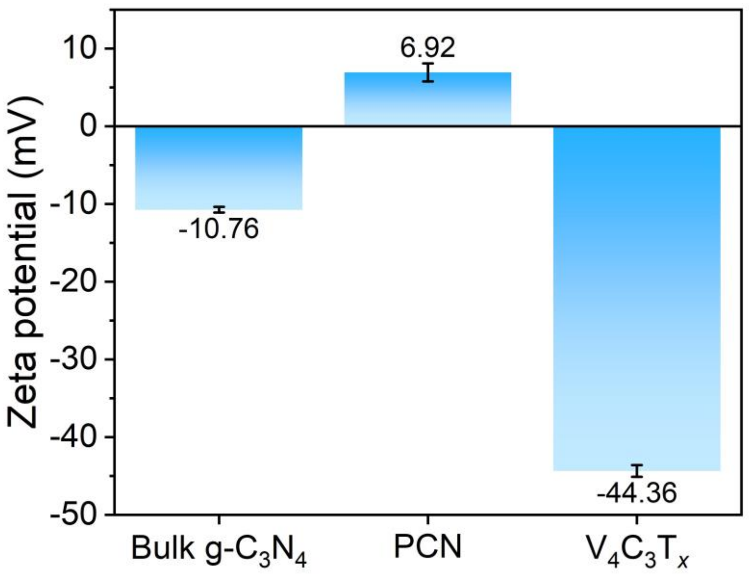

Figure S3. Zeta potential of bulk g-C_3N_4, PCN, and $V_4C_3T_x$ in aqueous solution.

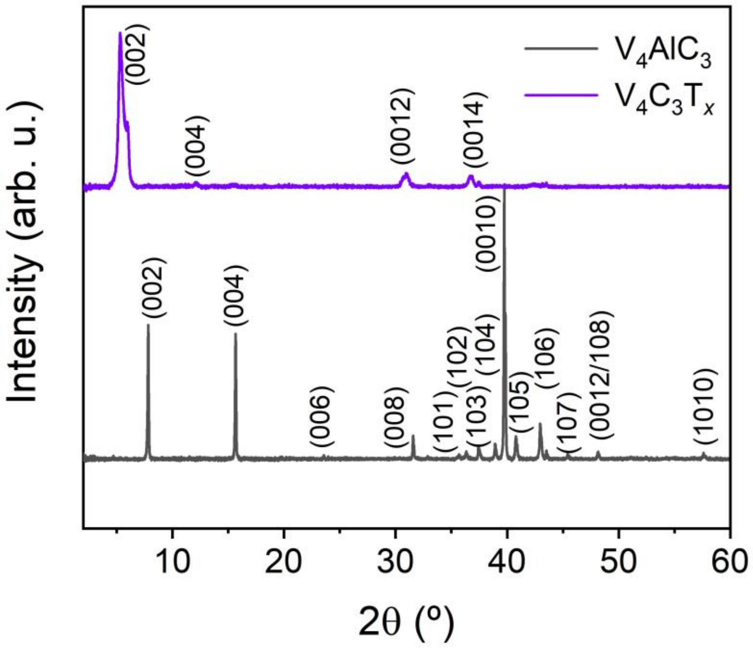

Figure S4. XRD pattern of V_4AlC_3 MAX and $V_4C_3T_x$ MXene.

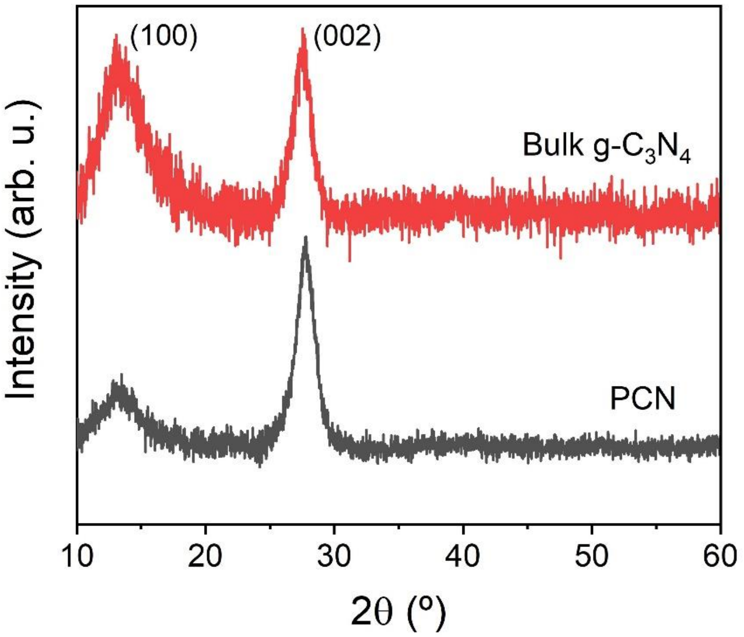

Figure S5. XRD spectra of bulk g-C_3N_4 and PCN.

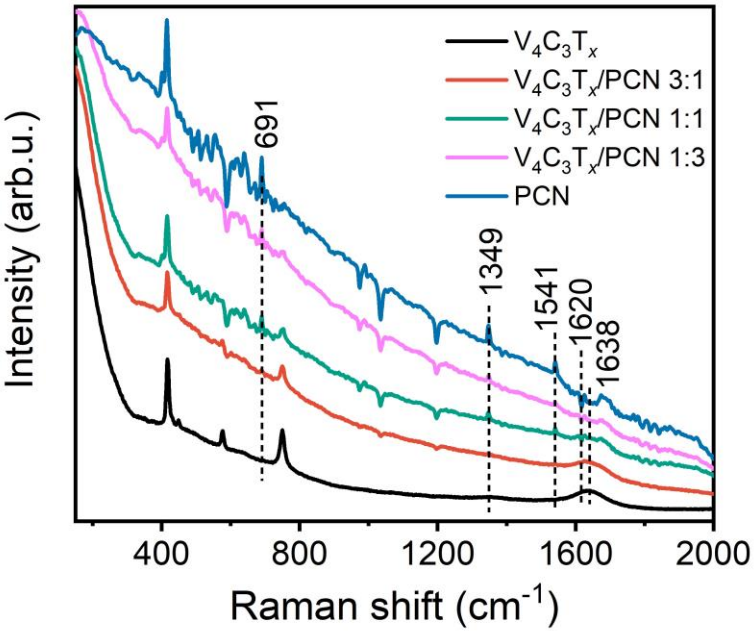

Figure S6. Raman spectra of PCN, $V_4C_3T_x$, and $V_4C_3T_x$/PCN heterostructures.

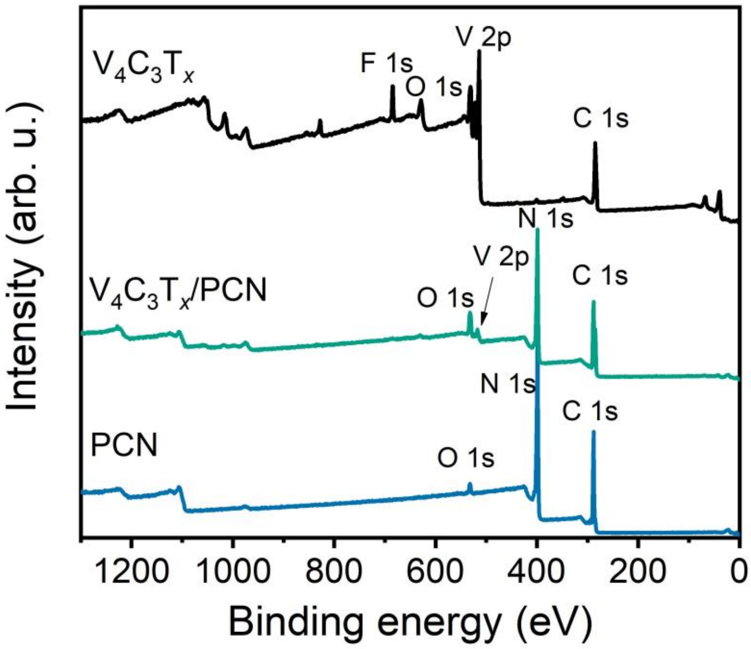

Figure S7. XPS survey spectra of PCN, $V_4C_3T_x$, and $V_4C_3T_x$/PCN 1:1 heterostructures.

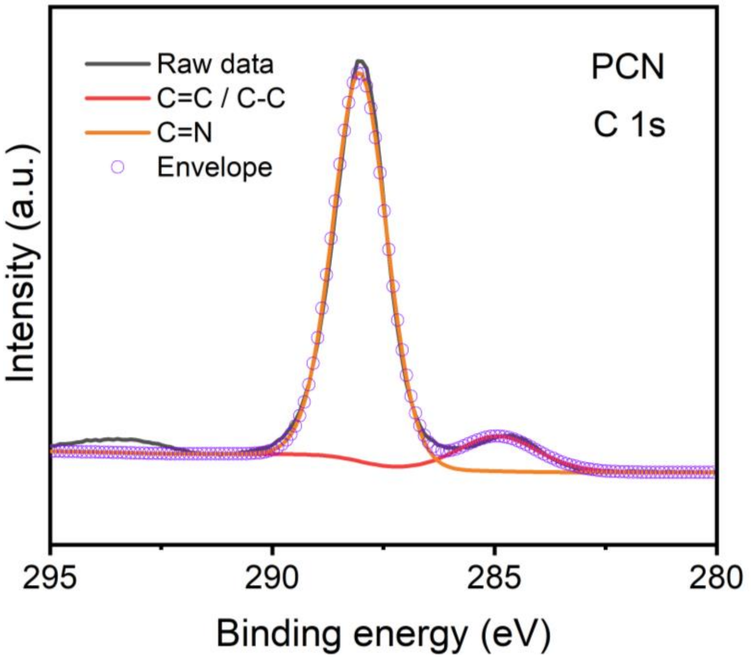

Figure S8. C 1s XPS spectra of PCN.

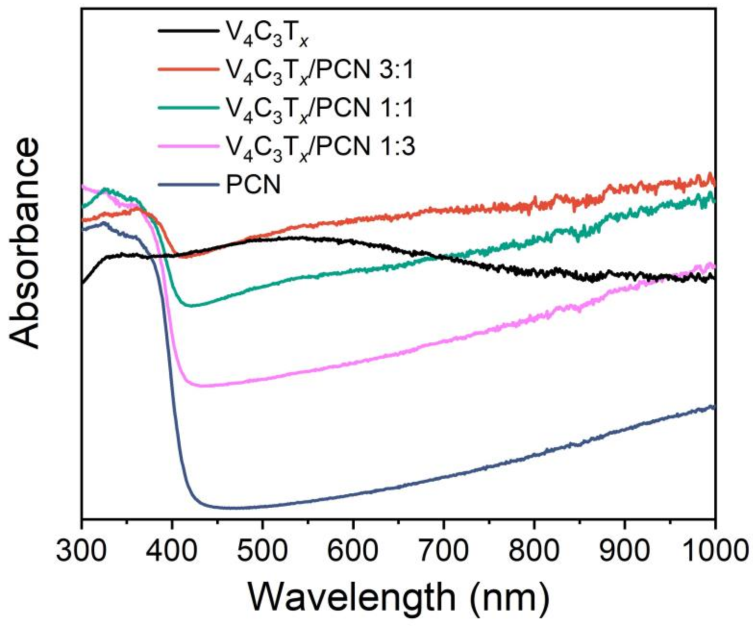

Figure S9. Absorption spectra of $V_4C_3T_x$, PCN, and $V_4C_3T_x$/PCN heterostructures obtained *via* UV-vis diffuse reflectance spectra.

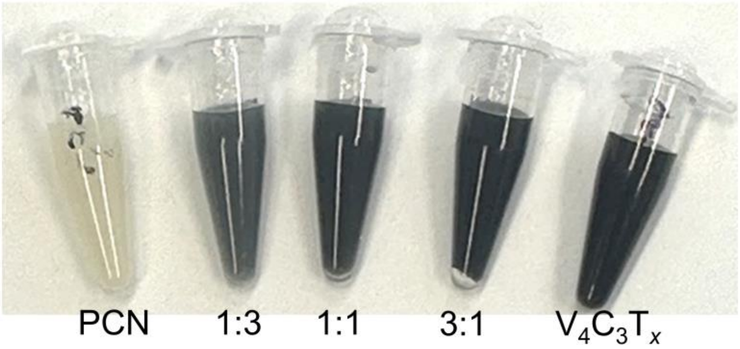

Figure S10. Digital photograph of aqueous dispersions of $V_4C_3T_x$/PCN heterostructures with a mass ratio for $V_4C_3T_x$:PCN of 1:3, 1:1, and 3:1 from left to right between PCN and $V_4C_3T_x$ vials at the two ends, showing the good colloidal stability.

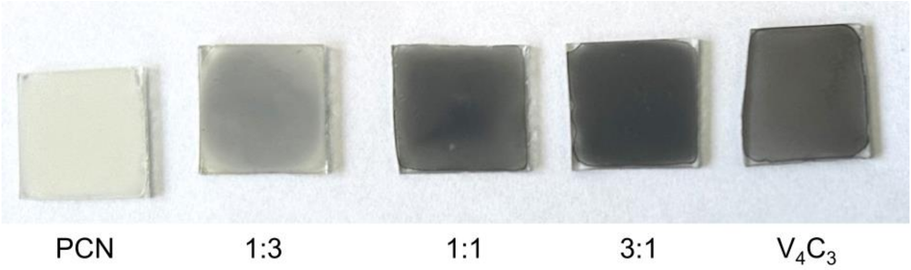

Figure S11. Digital photograph of the drop-cast thin films of $V_4C_3T_x$/PCN heterostructures with a mass ratio for $V_4C_3T_x$:PCN of 1:3, 1:1, and 3:1 from left to right between PCN and $V_4C_3T_x$ films at the two ends.

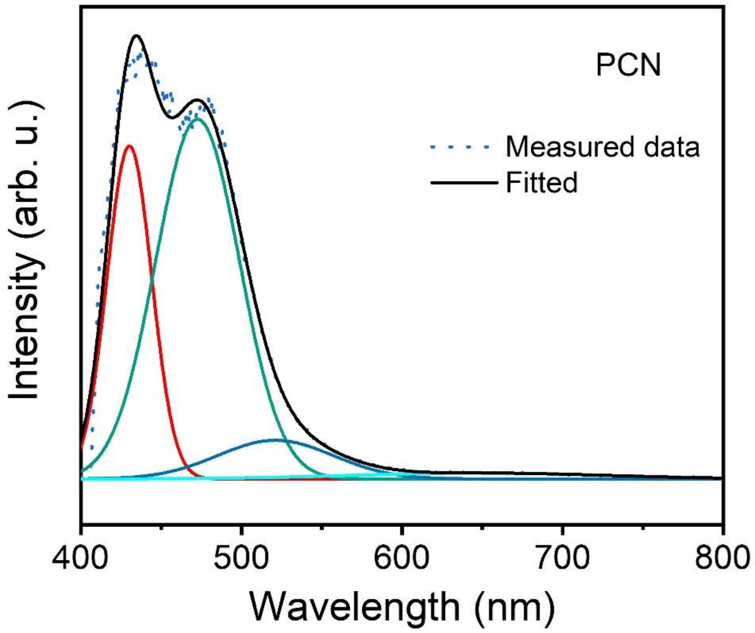

Figure S12. PL deconvolution of PCN at the measurement temperature of 270 K.

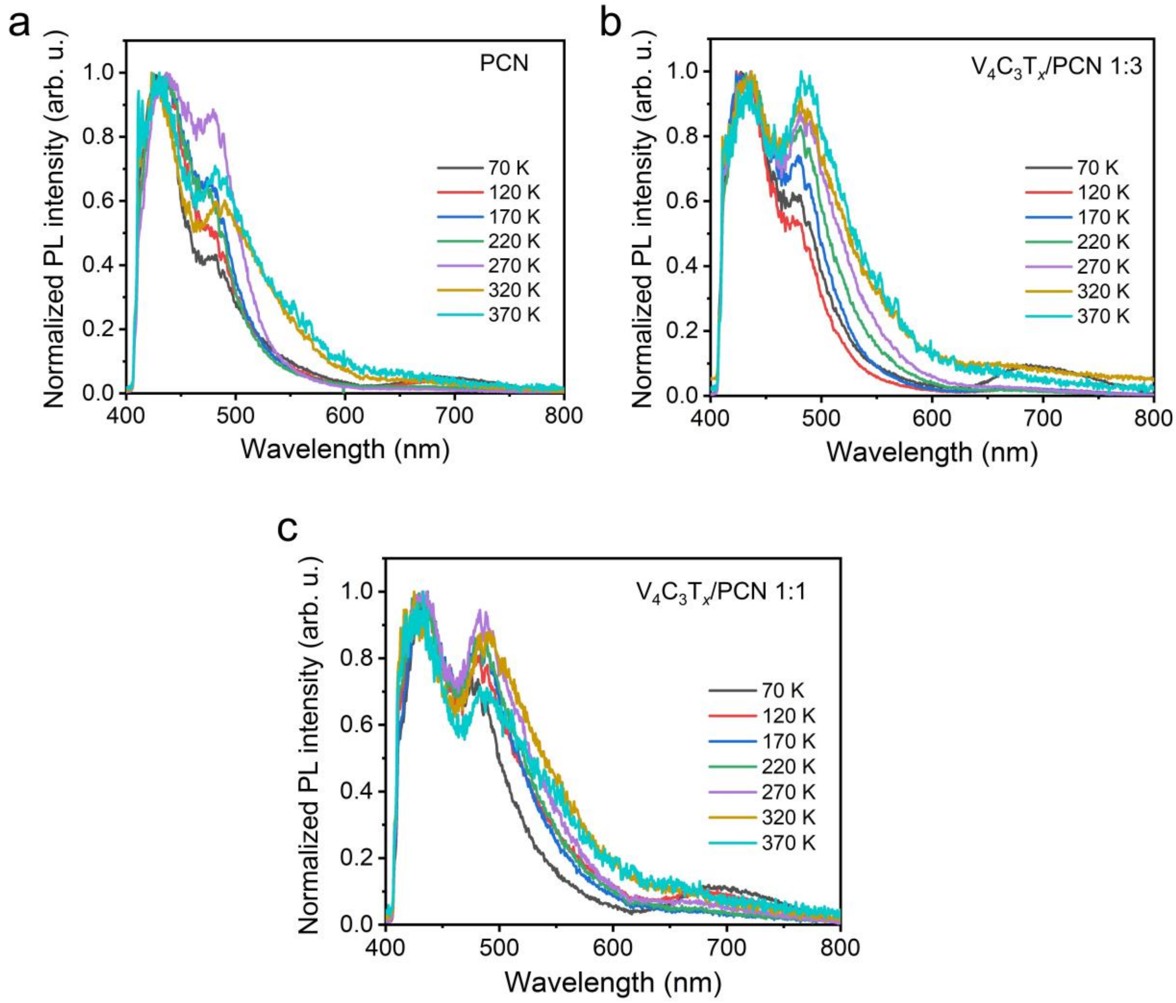

Figure S13. Temperature-dependent PL spectra of (a) PCN, (b) $V_4C_3T_x$/PCN 1:3, and (c) $V_4C_3T_x$/PCN 1:1.

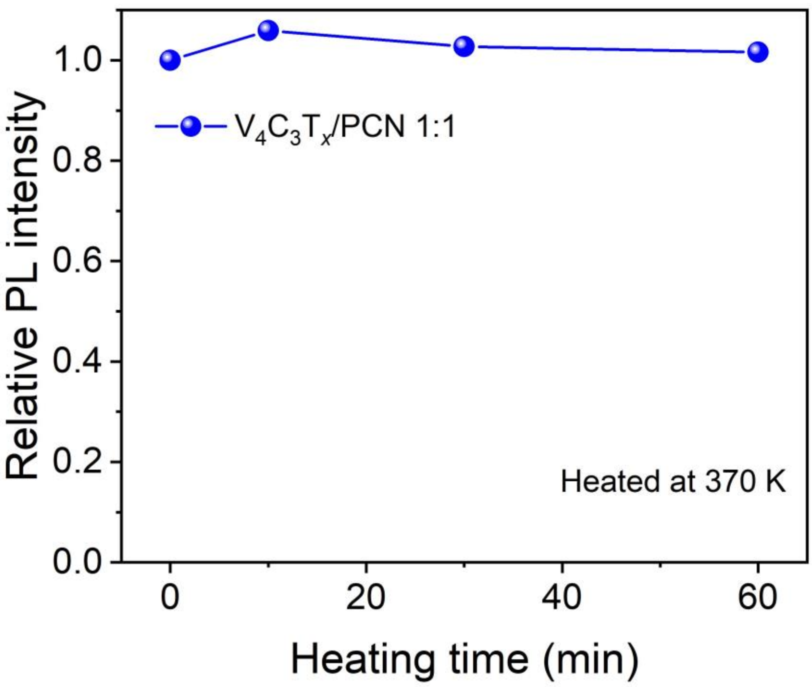

Figure S14. PL stability of $V_4C_3T_x$/PCN after performing heat treatment at 370K.

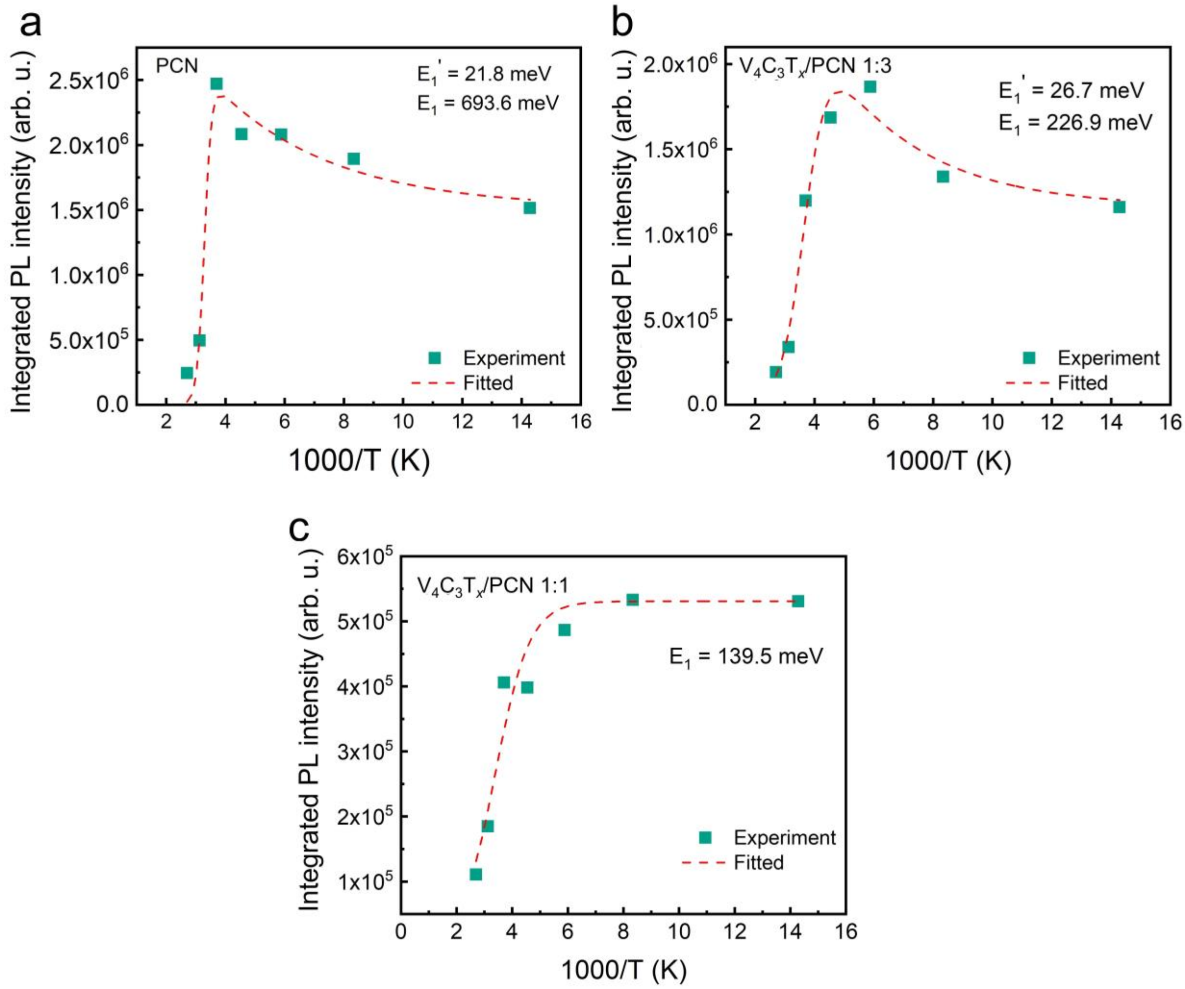

Figure S15. Thermal quenching fitting of (a) PCN, (b)$V_4C_3T_x$/PCN 1:3, and (c) $V_4C_3T_x$/PCN 1:1, by taking integrated area of 430 nm peak.

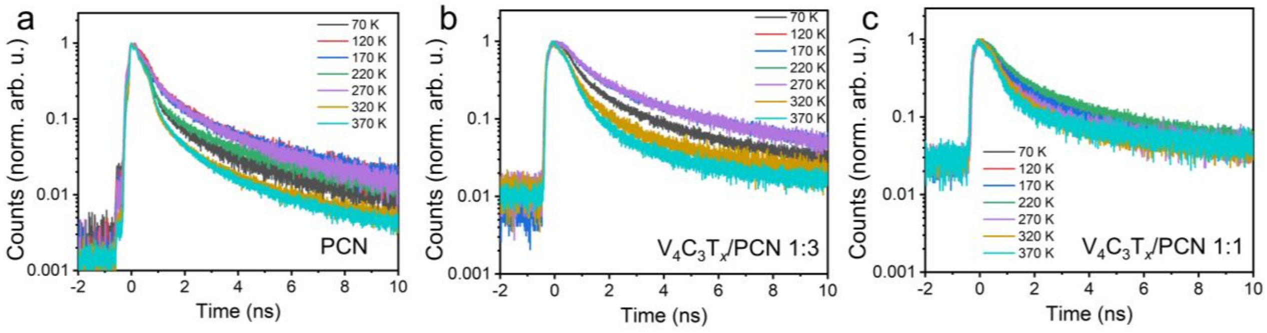

Figure S16. Comparison of time-resolved PL decay from (a) PCN, (b) $V_4C_3T_x$/PCN 1:3, and (c) $V_4C_3T_x$/PCN 1:1.

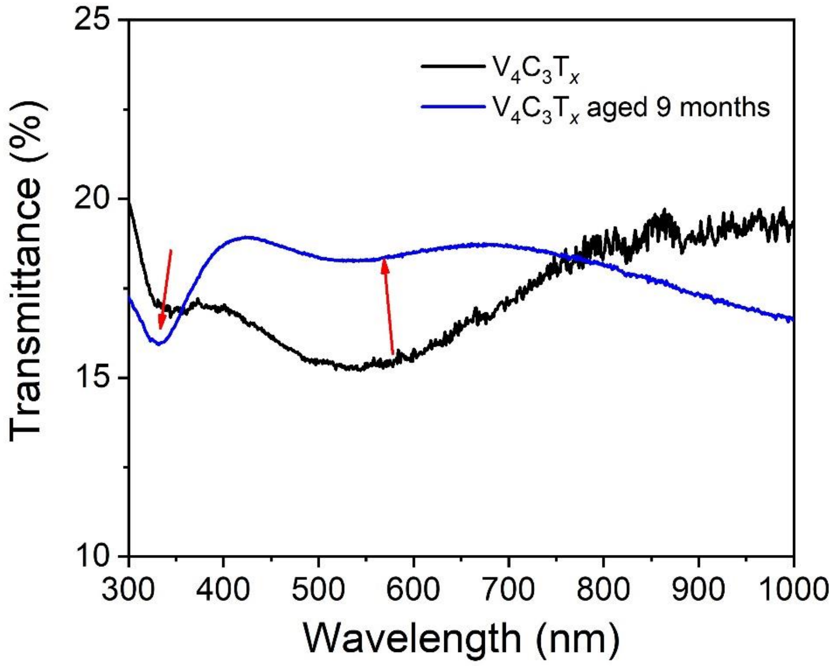

Figure S17. UV-Vis spectra of $V_4C_3T_x$ and $V_4C_3T_x$ aged 9 months. Here, after 9 months of aging the transmittance of $V_4C_3T_x$ increased in the visible region coupled with reduced transmittance in the UV region.

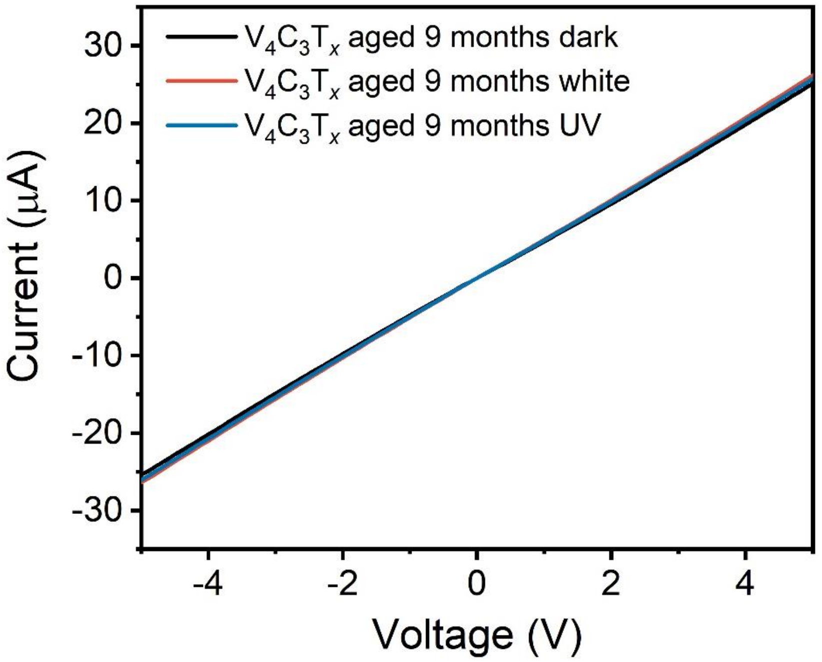

Figure S18. Current-voltage curve of $V_4C_3T_x$ aged 9 months in the dark and under white or UV light irradiation.

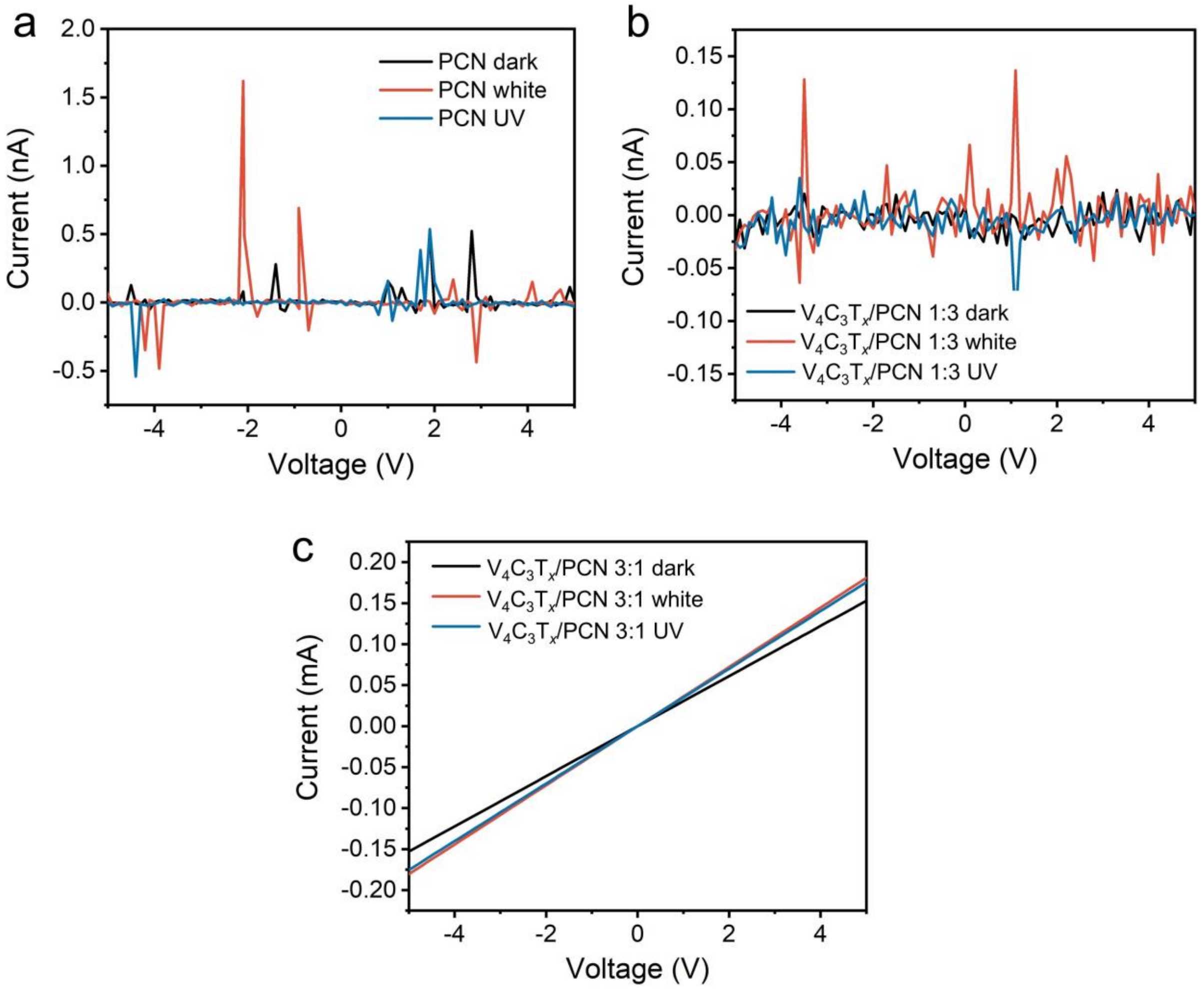

Figure S19. Current-voltage curve of (a) PCN, (b) $V_4C_3T_x$/PCN 1:3, and (c) $V_4C_3T_x$/PCN 3:1 heterostructures in the dark and under white or UV light irradiation.

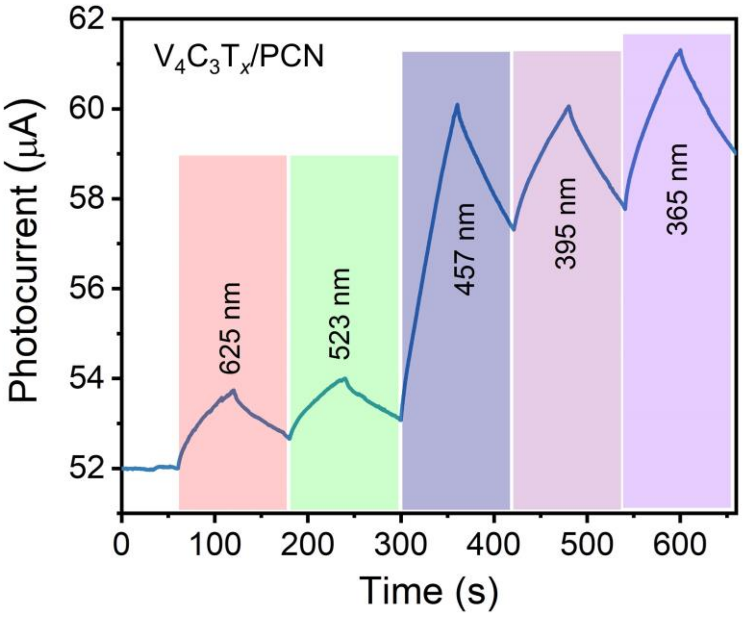

Figure S20. Photocurrent of $V_4C_3T_x$/PCN upon irradiation of different wavelengths. The bias voltage of 1.5 V was used.

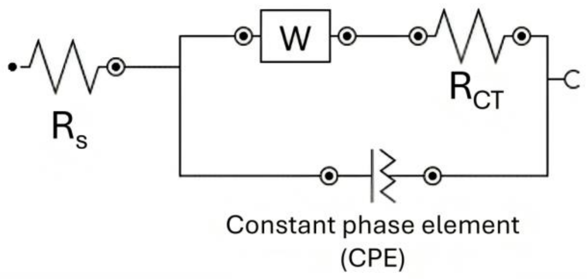

Figure S21. Equivalent Circuit Model for Electrochemical Impedance Spectroscopy (EIS) Fitting. Where, R_S is solution resistance, R_{CT} is charge transfer resistance, CPE is constant phase element, and W is the Warburg element.

Supplementary Tables

Table S1. Atomic concentration (%) of $V_4C_3T_x$, PCN, and $V_4C_3T_x$/PCN heterostructure obtained from XPS.

Sample	V 2p	C 1s	O 1s	F 1s	N 1s
PCN	-	44.63	3.21	-	52.15
$V_4C_3T_x$/PCN	3.17	48.25	6.27	-	42.31
$V_4C_3T_x$	18.19	55.49	17.21	9.11	-

Table S2. Fitting parameters of thermal quenching obtained by integrating excitonic peak.

Sample	A	A’	E (meV)	E’ (meV)
PCN	3.25×10^{11}	1.56	693.6	21.8
$V_4C_3T_x$/PCN 1:3	17.88×10^{3}	2.99	226.9	26.7
$V_4C_3T_x$/PCN 1:1	241.13	-	139.5	-

Table S3. Extracted fitting parameters of temperature-dependent TRPL for PCN. Where, τ_i is the decay time, (%) is the contribution of the decay time, and τ_{ave} is the average decay time.

Temperature (K)	τ_1 (ns)	τ_2 (ns)	τ_{ave} (ns)
70	0.49 ± 0.02 (66.1%)	4.56 ± 0.07 (33.9%)	1.88 ± 0.02
120	0.61 ± 0.03 (51.3%)	4.61 ± 0.11 (48.7%)	2.56 ± 0.04
170	0.59 ± 0.04 (51.1%)	4.74 ± 0.05 (48.9%)	2.71 ± 0.07
220	0.49 ± 0.02 (57.9%)	4.68 ± 0.08 (42.1%)	2.26 ± 0.09
270	0.60 ± 0.03 (57.9%)	4.16 ± 0.07 (42.1%)	2.39 ± 0.05
320	0.46 ± 0.04 (75.9%)	4.01 ± 0.13 (24.1%)	1.33 ± 0.06
370	0.45 ± 0.03 (76.1%)	3.94 ± 0.08 (23.9%)	1.29 ± 0.03

Table S4. Extracted fitting parameters of temperature-dependent TRPL for $V_4C_3T_x$/PCN 1:3. Where, τ_i is the decay time, (%) is the contribution of the decay time, and τ_{ave} is the average decay time.

Temperature (K)	τ_1 (ns)	τ_2 (ns)	τ_{ave} (ns)
70	0.92 ± 0.07 (55.6%)	6.61 ± 0.13 (54.4%)	3.45 ± 0.14
120	0.94 ± 0.02 (38.9%)	6.26 ± 0.21 (61.1%)	4.19 ± 0.17
170	0.95 ± 0.06 (55.6%)	6.31 ± 0.17 (54.4%)	4.23 ± 0.13
220	0.98 ± 0.10 (40.5%)	5.79 ± 0.12 (59.5%)	3.84 ± 0.09
270	1.01 ± 0.08 (41.4%)	5.83 ± 0.16 (58.6%)	3.83 ± 0.11
320	0.95 ± 0.06 (65.2%)	5.81 ± 0.09 (34.8%)	2.48 ± 0.17
370	1.03 ± 0.02 (74.6%)	5.88 ± 0.04 (25.4%)	1.99 ± 0.07

Table S5. Extracted fitting parameters of temperature-dependent TRPL for $V_4C_3T_x$/PCN 1:1. Where, τ_i is the decay time, (%) is the contribution of the decay time, and τ_{ave} is the average decay time.

Temperature (K)	τ_1 (ns)	τ_2 (ns)	τ_{ave} (ns)
70	0.72 ± 0.08 (57.5%)	5.90 ± 0.11 (42.5%)	2.92 ± 0.06
120	0.71 ± 0.03 (55.2%)	5.14 ± 0.14 (44.8%)	2.69 ± 0.10
170	0.85 ± 0.02 (51.7%)	5.88 ± 0.09 (48.3%)	3.28 ± 0.13
220	0.84 ± 0.04 (44.1%)	6.17 ± 0.16 (55.9%)	3.86 ± 0.07
270	0.71 ± 0.08 (56.5%)	5.79 ± 0.14 (43.5%)	2.92 ± 0.16
320	0.70 ± 0.05 (66.8%)	5.31 ± 0.14 (33.2%)	2.24 ± 0.07
370	0.59 ± 0.07 (56.5%)	6.37 ± 0.13 (43.5%)	3.02 ± 0.08

Table S6. Equivalent circuit parameters obtained from the EIS measurements.

Sample	R_s (Ω)	R_{CT} (Ω)	CPE (Y0)	CPE (N)	W (Mho.s$^{1/2}$)
PCN	16.17	27.58×10^3	3.59×10^{-5}	0.86	7.63×10^{-6}
$V_4C_3T_x$/PCN 1:3	15.37	14.50×10^3	7.28×10^{-5}	0.88	2.32×10^{-4}
$V_4C_3T_x$/PCN 1:1	13.12	11.02×10^3	9.41×10^{-5}	0.88	2.62×10^{-4}
$V_4C_3T_x$/PCN 3:1	13.41	4.88×10^3	1.10×10^{-4}	0.88	4.92×10^{-4}
$V_4C_3T_x$	14.39	3.59×10^3	9.02×10^{-5}	0.89	1.26×10^{-3}

References

(1) Shibata, H. Negative Thermal Quenching Curves in Photoluminescence of Solids. *Japanese Journal of Applied Physics* **1998**, *37* (2R), 550. https://doi.org/10.1143/JJAP.37.550.
